# Outcomes of Experiencing Interpersonal Violence in Autism: A Mixed Methods Systematic Review and Meta-Analysis

**DOI:** 10.1177/15248380251357618

**Published:** 2025-08-09

**Authors:** Kassandrah Cooke, Kathryn Ridgway, Laura Pecora, Elizabeth Westrupp, Darren Hedley, Merrilyn Hooley, Mark A. Stokes

**Affiliations:** 1 Deakin University, VIC, Australia; 2 Deakin University, Burwood, Australia; 3 La Trobe University, VIC, Australia

**Keywords:** autism, interpersonal violence, mental health, suicide, gender, systematic review, meta-analysis

## Abstract

In this review and meta-analysis, we aimed to examine outcomes of interpersonal violence among autistic people. Intersectionality and minority theories suggest that negative outcomes are heightened among people with multiple marginalized identities. Thus, we also aimed to investigate gender-related outcomes of interpersonal violence among autistic people. We conducted a systematic database search with inclusion criteria including mixed methods, peer-reviewed research examining any harmful interpersonal act (e.g., physical, sexual, and psychological) experienced by autistic people. We undertook a random-effects meta-analysis with pooled data from 9 studies, comprising 3,647 autistic participants aged 1 to 80 years. Violence was associated with worsened mental health, with the strongest association for internalizing symptoms (*d* = 0.66, *p* < .001; 95% CI [0.51, 0.80]) and suicidal thoughts and behavior (*d* = 0.63, *p* < .001; [0.44, 0.82]). Narrative synthesis of 57 studies comprising 37,418 participants (13,127 autistic, 24,291 non-autistic) found violence was associated with numerous adverse health, development, and functional outcomes, including worsened mental health and behavioral difficulties compared to non-autistic controls from childhood. Females and gender minorities reported greater intra- and interpersonal health and development difficulties related to violence, emerging in early childhood and enduring into adulthood. Findings provide strong evidence of lifelong negative outcomes associated with interpersonal violence experienced by autistic people, providing evidence for the relevance of minority stress and intersectionality theories in understanding risk. Indeed, our results raise concerns that autistic people, and particularly non-male (female, gender diverse) individuals, have higher susceptibility for abuse from a young age, while being conditioned to respond with social desirability, superficial adaptivity, and dissociation.

Approximately half of the autistic population^1^ experience poorer outcomes in securing employment, having social or romantic relationships, and living independently than those in the broader population (Hancock et al., 2017; Howlin, 2021; Mason et al., 2021; Pecora et al., 2016; Steinhausen et al., 2016). Of particular concern are the increased mental health challenges (e.g., depression, anxiety, self-harm, suicidal thoughts and behavior) observed among autistic people, relative to non-autistic people, with these challenges even more apparent among autistic females and gender diverse people (Brown et al., 2024; Santomauro et al., 2024; Simpson et al., 2024). Identifying factors that may negatively influence optimal health and functioning in the autistic population is imperative for developing appropriate support strategies, yet it poses an impactful challenge for researchers. However, emerging evidence highlights the importance of examining the contribution of interpersonal violence to negative outcomes, given the increased risk of violence experienced by autistic people (Cooke et al., 2024; Pecora et al., 2016; Trundle et al., 2022).

Interpersonal violence refers to violence that occurs between individuals, including family members (i.e., domestic violence; American Psychological Association, 1996), intimate partners (i.e., intimate partner violence; Centers for Disease Control and Prevention [CDC], 2021), and members of a community (Krug et al., 2002). The World Health Organization (WHO) conceptualizes interpersonal violence as involving the intentional use of physical force or abuse of power (Dahlberg & Krug, 2002; Krug et al., 2002) that includes harmful or cruel acts of a physical, sexual, or psychological nature, as well as neglect or acts of omission (Krug et al., 2002; Mercy et al., 2017; Olweus, 1993). These acts are defined by their resulting in, or high likelihood of resulting in, physical, psychological, or developmental harm, death, or deprivation, irrespective of the intentionality of the outcome (Dahlberg & Krug, 2002; Krug et al., 2002). Interpersonal violence is a widely demonstrated threat to public health and well-being (James et al., 2020; Rosenberg et al., 2006; Sethi & Peshevska, 2014). The effects of interpersonal violence impact families and communities across generations, globally (Mercy et al., 2017), incurring substantial economic, social, emotional, and well-being costs to individuals and society. These include costs associated both with prosecuting perpetrators and treating those victimized (Krug et al., 2002), in terms of long-term health outcomes, such as increased psychopathology in adulthood (Kessler et al., 2010), and associated impacts on academic, employment, and relational functioning (Shapland et al., 1985). Despite consistent findings highlighting the detrimental impact of violence on mental health and functioning in the general population, the effects of violence among autistic populations have, until recently, been largely overlooked.

Emerging research evidence suggests the harmful impacts of violence may be exacerbated among autistic populations. Notably, autistic people have a heightened individual and social risk of experiencing various forms of violence across the life course than the general population (Cooke et al., 2024), and a heightened susceptibility toward developing their sequelae (e.g., post-traumatic stress disorder [PTSD], non-suicidal self-injury, suicidal thoughts and behavior; see Fuld, 2018). Such challenges occur alongside a lack of public awareness, understanding, and accessibility geared toward autism, presenting barriers for autistic people to access appropriate intervention (Adams & Young, 2020; Malik-Soni et al., 2022; Mason et al., 2019) and criminal justice support (Archer & Hurley, 2013; Chown, 2010; Cooper et al., 2022). A systematic and quantitative synthesis of available literature on the associated impacts of violence in autism across individual (e.g., mental health, non-suicidal self-injury, suicidal thoughts and behavior) and social contexts (e.g., relationships, employment, community involvement; Bronfenbrenner, 1977, 1989) could therefore provide necessary insights for violence prevention and support efforts.

Exposure to interpersonal violence (i.e., experiencing or witnessing) may also result in detrimental consequences that vary at different life-course stages (Elder, 1994; Olofsson, 2014). For example, adverse childhood experiences (ACEs) encompassing violent events (e.g., maltreatment or domestic violence exposure) during childhood or adolescence (Hughes et al., 2017), can influence the incidence of future violence against the individual, and play a role in shaping health-related outcomes (CDC, 2020; Hughes et al., 2017). In the general population, ACEs are associated with numerous adverse psychological, emotional, and social outcomes (Haahr-Pedersen et al., 2020), with exposure to multiple childhood adversities being associated with an elevated risk of detrimental health outcomes throughout one’s life (see Hughes et al., 2017). Such findings highlight the pervasive and detrimental impact that interpersonal violence exposure can have during critical developmental periods. However, the ways that such experiences impact autistic people throughout the life course are unknown.

The detrimental impacts of violence may further intersect with gender, such that autistic females and gender minorities (e.g., transgender and gender queer) may be particularly affected (e.g., Moseley et al., 2021, 2024). Gender is a multidimensional construct that encompasses identity, roles, and experiences, which are shaped by psychological, biological, social, and cultural factors (Mazzuca et al., 2020). Distinct from sex, gender extends beyond binary categorizations of male and female and acknowledges a diverse spectrum of identities that are influenced by these factors (Mazzuca et al., 2020). Under the minority stress model (Meyer, 2003), people with a minority identity (e.g., being autistic) experience additional social stressors (e.g., discrimination and violence), which constitute risk factors for worsened mental health outcomes compared to broader populations (Frost et al., 2015; Lewis et al., 2003; Meyer, 2003). In the general population, there is evidence that females and gender minorities experience increased risk of intimate partner or sexual violence and its sequelae (Brown & Herman, 2015; Johns et al., 2019; Sardinha et al., 2022). Such stressors are suggested to be heightened among people with *multiple* marginalized identities (Meyer, 2003), such as being autistic *and* female (e.g., Dowse et al., 2016; Grollman, 2014). Supporting this theory, there is evidence showing that autistic females and gender minorities present with increased risks of experiencing various forms of violence than autistic cis-males (Cooke et al., 2024; Pecora et al., 2016), with the post-traumatic effects of violence exposure appearing particularly pronounced among autistic gender marginalized groups (Haruvi-Lamdan et al., 2020; Reuben et al., 2021). So considered, a systematic integration of available literature on the impacts of violence among autistic people of varying gender identities is warranted.

The current systematic review and meta-analysis aimed to examine individual and social impacts of violence throughout the life course and investigate violence outcomes as they relate to gender among autistic people. Previous reviews examined the relationship between victimization and experiencing additional forms of violence among autistic boys and girls (Hellstrom, 2019) and sexual violence outcomes among autistic people who varied in age, gender, and level of support needs (Dike et al., 2022). Research findings to date indicate a relationship between victimization and experiencing additional forms of violence and mental health challenges (e.g., internalizing symptomology, non-suicidal self-injury, suicidal thoughts and behavior, behavioral challenges) among autistic people. However, there is a need to clarify these associations while considering intersectionality in relation to individual (e.g., autism characteristics, mental health, behavior, and gender) and social sequelae (e.g., service use and repeated victimization) associated with varying forms of violence experiences across the life course. This review aimed to: (a) thematically and quantitatively synthesize evidence on the relationship between interpersonal violence and individual (e.g., mental health and behavior) and social (e.g., access to support and stigma) outcomes among autistic people. These were contextualized by developmental stage (e.g., childhood, adolescence, and adulthood). (b) Examine how these outcomes might intersect with gender-related trends to shape vulnerability toward violence outcomes.

## Method

### Protocols and Registration

We registered this systematic review with the International Prospective Registry of Systematic Review in July 2021, registration number: CRD42021252481. The search strategy formed part of a larger systematic review on autistic experiences of interpersonal violence, with three components. The first component entailed a narrative synthesis of individual, social, and life-course risk factors associated with interpersonal violence among autistic populations. The second component involved a narrative synthesis and meta-analytic examination of gender-related differences in interpersonal violence prevalence among autistic people. The current review examines the association between violence experiences and outcomes across individual, social, and gender-related domains across developmental stages among autistic people, combining meta-analytic and thematic synthesis methods. This review adheres to PRISMA guidelines (Liberati et al., 2009).

### Study Eligibility Criteria

We undertook a systematic search of available articles examining autistic experiences of interpersonal violence. We included all research studies if their findings included outcomes in relation to interpersonal violence exposure (being witness to or subject to) among participants; studies examining risk factors of violence were excluded. Rather than restricting outcomes to predefined categories, we coded and grouped them thematically during data extraction and synthesis into individual (e.g., mental health, well-being, and behavior) and social (e.g., relationships, service use, and community assistance) domains. Utilizing the WHO’s conceptualization of interpersonal violence, violence was defined as any violent act (e.g., physical, sexual, and psychological) that occurred interpersonally and was associated with harm (Dahlberg & Krug, 2002; Krug et al., 2002). This definition was then cross-referenced with study-level definitions or descriptions of violence experiences. Studies were included if participants were formally diagnosed or self-identified as autistic and were not all of one sex or gender (e.g., the entire sample reported or identified as female) to allow for consistency of intersectional comparisons across gender groups for each outcome(s) examined in each study. This aligned with the current review’s investigative focus on gender-related outcomes, conceptualized in this review as incorporating both sex- and identity-based experiences, derived from study-level data. We placed no restrictions on participant age or support needs level (e.g., co-occurring intellectual disability [ID]). We included studies that used reliable and validated instruments to measure interpersonal violence, as determined via review of the instruments’ psychometric properties and prior use in research; however, studies that used alternative measures, including novel questionnaires, parent-report measures, and qualitative interviews, were also included. We excluded these: opinion pieces, reviews, editorials, commentaries, animal studies, conference abstracts, and case studies, as they did not contain primary research data or were not peer-reviewed. Studies that did not test gender-related differences or provide sufficient data to permit data extraction for our analysis were also excluded. We did not impose restrictions on language or publication date regarding study eligibility.

### Information Sources

We conducted a systematic search of Excerpta Medica Database, MEDLINE Complete, Cumulative Index of Nursing and Allied Health Literature, PsycINFO, SocINDEX, and Web of Science. We also screened reference lists of included studies to screen for additional studies of relevance. This resulted in including two additional studies. Our search syntax included synonyms for: (a) autism; (b) interpersonal violence; and (c) participant sex (male, female) or gender (e.g., transgender and nonbinary). The first author conducted an initial database search in June 2021 and an updated search in January 2024. The electronic search strategy of the reported databases is shown in Table S1, while information on study selection and data extraction is listed in Supplemental Information: Expanded Methods.

### Summary Measures and Statistical Analysis

To examine the association between the likelihood of experiencing interpersonal violence and individual (e.g., mental health, self-esteem, and behavior) and social outcomes (e.g., proximal social, community, and societal), we conducted subgroup analyses on each violence outcome examined by more than two studies of cross-sectional designs, given their ability to explore multiple outcomes (Mann, 2003). A random-effects model was chosen to address issues with detecting heterogeneity due to low power (Liberati et al., 2009).

We implemented a convergent segregated approach for synthesizing and integrating data, whereby quantitative and qualitative data are analyzed separately, then integrated (see Stern et al., 2020). Regarding data extraction, we identified study data that reported violence exposure likelihood or frequency, for recent or lifetime victimization, and its association with various individual and social outcomes, and included this in the syntheses. For the purposes of this review, we defined *recent* victimization as occurring within the past 12 months, as indicated by study-level reporting. We extracted information regarding study design (e.g., cross-sectional and longitudinal) where reported. Studies that examined quantitative relationships between violence exposure and outcomes that were sufficiently comparable (e.g., measured by at least two studies) were eligible for inclusion in the meta-analysis. All remaining studies examining quantitative associations that were not sufficiently comparable (e.g., less frequently assessed), as well as qualitative accounts, were included in our thematic analysis. For studies retained for meta-analysis, we converted each effect size that was extracted from the included studies to standardized mean group difference (Cohen’s *d*, [95% CI]; Cohen, 1988) or computed effect sizes that were not reported via the data provided. The strength of effect sizes ranged from small (*d* = 0.20), moderate (*d* = 0.50), to large (*d* = 0.80; see Cohen, 1988). The effect sizes of exposure to one or more violence experiences and their association with all outcomes among participants with or without an abuse history were then pooled utilizing the inverse variance method using Review Manager, Version 5.1.1 (The Cochrane Collaboration, 2020). Heterogeneity was interpreted via Cochran’s *Q* and *I*^2^ statistics.

In accordance with the convergent segregated approach detailed above (Stern et al., 2020), all remaining data—including qualitative and extracted quantitative data that did not meet meta-analysis requirements, were integrated into a thematic synthesis. Specifically, all quantitative data were transformed via conversion into qualitative data (i.e., data were *qualitized*; see Stern et al., 2020) and reported via a comprehensive thematic synthesis in which we grouped findings across violence types (e.g., physical, sexual, and psychological) and into individual (e.g., mental health, behavior) and social outcome domains (e.g., relationships and community support). We then further contextualized these domains across life stages (e.g., childhood, adolescence, and adulthood) and by gender-related patterns. We extracted information pertaining to participant age, sex, and gender from study-level data, with some reporting gender identity (e.g., gender fluid and transgender), and others only reporting binary sex (male, female). These data were recorded accordingly, and *gender-related* terms were used to denote group differences and identity-informed variations throughout the synthesis. The reported, condensed thematic synthesis reflects core themes identified across individual and social outcome domains, drawing from a comprehensive thematic synthesis of these domains as detailed in Supplemental Information: Expanded Results. Participant characteristics and study details are reported in Table 1.

**Table 1.** Participant Demographic Information.

	Meta-Analysis		Systematic Review	
	*n* = 3,647		*n* = 37,418	
Participant Characteristics	No. (%)	*M* (*SD*)	No. (%)	*M*(*SD*)
Autism Status				
Autistic	3,647 (100)	—	13,127 (35.1)	0.35 (92.3)
Non-autistic	0 (0)	—	24,291 (64.9)	0.65 (92.3)
Gender				
Male	2,406 (6)	0.66 (28.6)	21,206 (56.7)	0.57 (95.9)
Female	886 (24.3)	0.24 (25.9)	15,600 (41.7)	0.42 (95.4)
Gender minorities	260 (7.1)	0.07 (15.5)	345 (0.9)	0.009 (18.5)
Other/n/s^a^	95 (2.6)	0.03 (9.6)	267 (0.7)	0.007 (16.2)
Race				
White	2,347 (64.4)	0.64 (28.9)	27,348 (73.1)	0.73 (85.7)
Black	131 (3.4)	0.04 (11.3)	419 (1.1)	0.01 (20.4)
Asian	804 (22)	0.22 (25.1)	859 (2.3)	0.02 (29.0)
Hispanic	79 (2.2)	0.02 (8.7)	98 (0.3)	0.003 (9.9)
Native American	13 (0.4)	0.004 (3.6)	18 (0.05)	0.0005 (4.3)
Mixed race	5 (0.1)	0.001 (2.3)	56 (0.1)	0.002 (7.5)
Non-white/other	149 (4.1)	0.04 (12.0)	4,877 (13)	0.13 (65.3)
n/s	119 (3.3)	0.03 (10.7)	3,743 (10)	0.10 (58.0)
Victimized				
Yes	2,006 (55)	0.55 (30.0)	—	—
No	1,641 (45)	0.45 (30.0)	—	—
Autistic Participants with ID				
Yes	489 (13.4)	0.13 (20.6)	1,637 (12.5)	0.12 (37.7)
No	2,289 (62.8)	0.63 (29.3)	7,384 (56.3)	0.56 (56.7)
n/s	869 (23.8)	0.24 (25.7)	4,107 (31.3)	0.31 (53.0)

*Note.* ^a^n/s = Nonspecified. ID = intellectual disability.

### Study Selection

**Figure 1.**
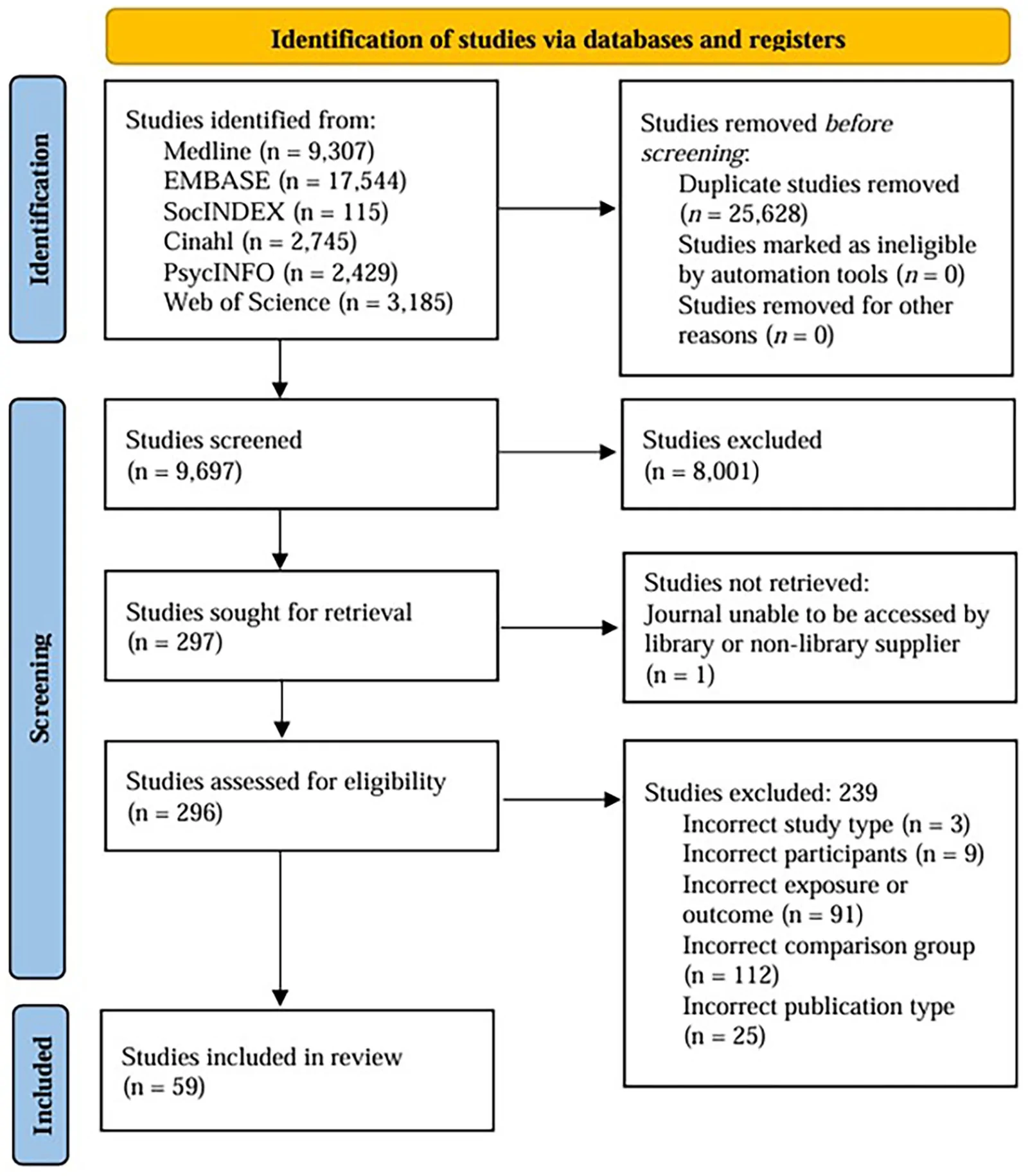
PRISMA 2020 flowchart for study inclusion. *Source.* Page et al. (2021).

We identified 35,325 studies in the preliminary database search, removed 25,628 duplicates, and identified 296 studies for full-text screening. Any disagreements regarding study eligibility were resolved by discussion. This was only required for three articles. We retained 59 studies for inclusion (Figure 1).

### Study Characteristics: Methods and Participants

Of all 59 studies included in the review, 9 observational and cross-sectional studies, published between 2013 and 2022, were included in the quantitative synthesis examining outcomes of violence in autism among 3,647 autistic participants (55% victimized, 45% not victimized), aged between 1 to 80 years. Participants were reported to be formally diagnosed with autism or self-identified as autistic. Data were obtained for all nine studies via: four self-report, three parent-report, one parent- and self-report, and one parent- and clinician-report measures.

Data not included in the meta-analysis were examined via qualitative synthesis of 57 of 59 articles, with two articles (Chiu et al., 2018; Chou et al., 2020) not included as they provided no new data beyond those examined in the meta-analysis. Studies were published between 2005 and 2023, and used qualitative (*n* = 11), quantitative (*n* = 37), and mixed methods designs (*n* = 9). These studies included a total of 13,127 autistic participants and 24,291 non-autistic participants. Data were obtained via 16 self-report, 10 parent-report, 6 self- and parent-report, 1 peer-, teacher- and parent-report, 1 peer- and parent-report, 1 parent- and clinician-report, 2 self- or parent- and teacher-report, 13 semi-structured or structured interviews, 2 open-ended surveys, and 3 clinical records. A detailed description of participants’ characteristics and assessment of individual- and social-level outcomes examined by each study can be made available on request.

### Study Quality

We assessed study quality with the Mixed Methods Appraisal Tool (MMAT; Hong et al., 2018), developed to appraise the quality of qualitative, randomized controlled, nonrandomized, quantitative descriptive, or mixed methods designs. We summarized the study quality of each included study, rather than excluding studies of low methodological quality, which is discouraged (Hong et al., 2018).

## Results

### Study Quality

Among all 59 studies, 33 met 4 or 5 of the MMATs criteria for each study design and so were rated as high quality (Table S3). However, they had challenges relating to the limited generalizability of findings derived from participants who tended to be white, male, without ID, and/or were from higher-income backgrounds. Additional issues related to potential selection bias (e.g., participants who experienced violence may have been more likely to participate) and measurement bias (e.g., using data not intended for research purposes, data subject to inaccuracies). There were also issues relating to data collection, including sole reliance on self- or parent-report data (e.g., limits nuanced investigations, violence may be under-reported or under-estimated, recall bias, misinterpretation). Twenty-two studies rated as good quality had similar but additional limitations related to mixed method reporting (e.g., inadequate rationale for study design, lack of integration/contrasting of mixed methods data) and using measurement tools not validated for autistic use. They also reported statistical issues, including inadequate power due to small samples, and risk of non-response bias (i.e., low parent inclusion rate) and confounder bias (e.g., age not homogenous between groups). Six studies were rated as low quality due to potential selection bias, measurement bias, and statistical issues, such as an unreliable statistical model, missing data, and small sample size.

### Risk of Bias Across Studies: Publication Bias

We observed significant (*p* < .05) and high levels of heterogeneity in the meta-analysis examining associations between interpersonal violence and mental health and behavioral challenges, *I*^2^ = 93% (lifetime, *I*^2^ = 0.60%; past year, *I*^2^ = 0.94%). Inspection of a funnel plot suggested asymmetry, further indicating publication bias (Figure S1). Given our attempts to include all articles of relevance, this level of heterogeneity may be attributed to the few studies included in our analysis (*n* = 9), as well as variability in age groups and instruments used to measure violence and associated outcomes. Accordingly, we suggest our quantitative synthesis be included but interpreted with caution.

### Study Characteristics: Exposures and Outcomes

We conducted a meta-analytic examination of the likelihood that a history of experiencing violence (e.g., one or more forms of past year or lifetime violence) would be associated with experiencing various individual (e.g., mental health, well-being, and behavior) or social outcomes (e.g., proximal social, community, and societal) among autistic people. Nine studies met criteria for meta-analysis, which included studies that primarily examined exposure to multiple forms of violence (e.g., physical, sexual, and psychological), with some also assessing bullying or cyberbullying. These studies also only examined individual-level outcomes relating to mental health and behavioral challenges. We utilized subgroup analyses to examine each reported outcome associated with violence explored by two or more studies. These included: (a) internalizing symptoms (e.g., depression, anxiety, OCD, and insomnia); (b) externalizing challenges (e.g., hyperactivity/impulsivity, opposition, and inattention); (c) Post-traumatic Stress Symptomology (PTSS; e.g., intrusion symptoms, avoidance, and alterations in arousal/reactivity); (d) suicidal thoughts and behavior (e.g., suicidal ideation and attempted suicide); and (e) self-esteem.

### Synthesis of Results

**Figure 2.**
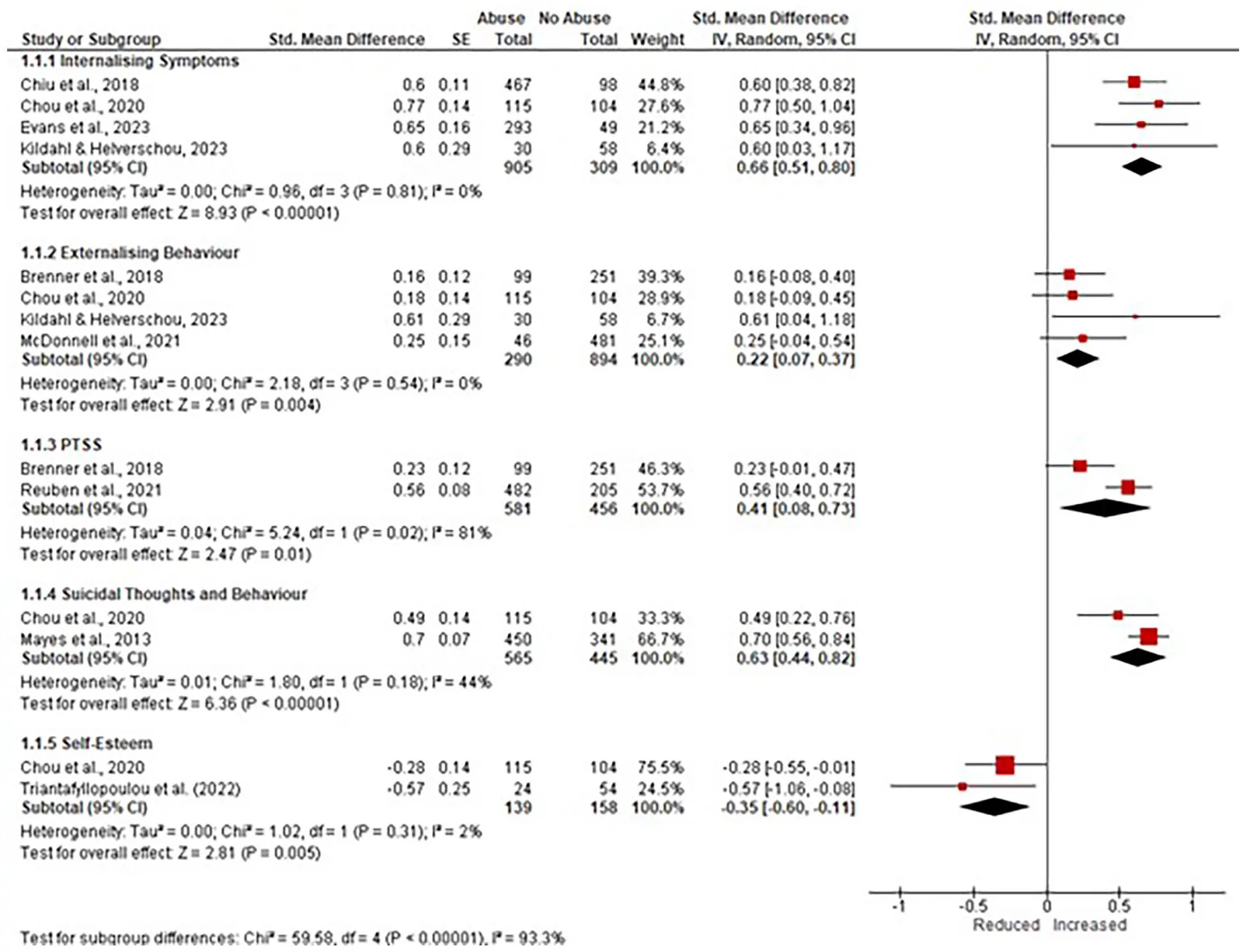
Forest plot of associations between violence and mental health and behavioral factors.

**Figure 3.**
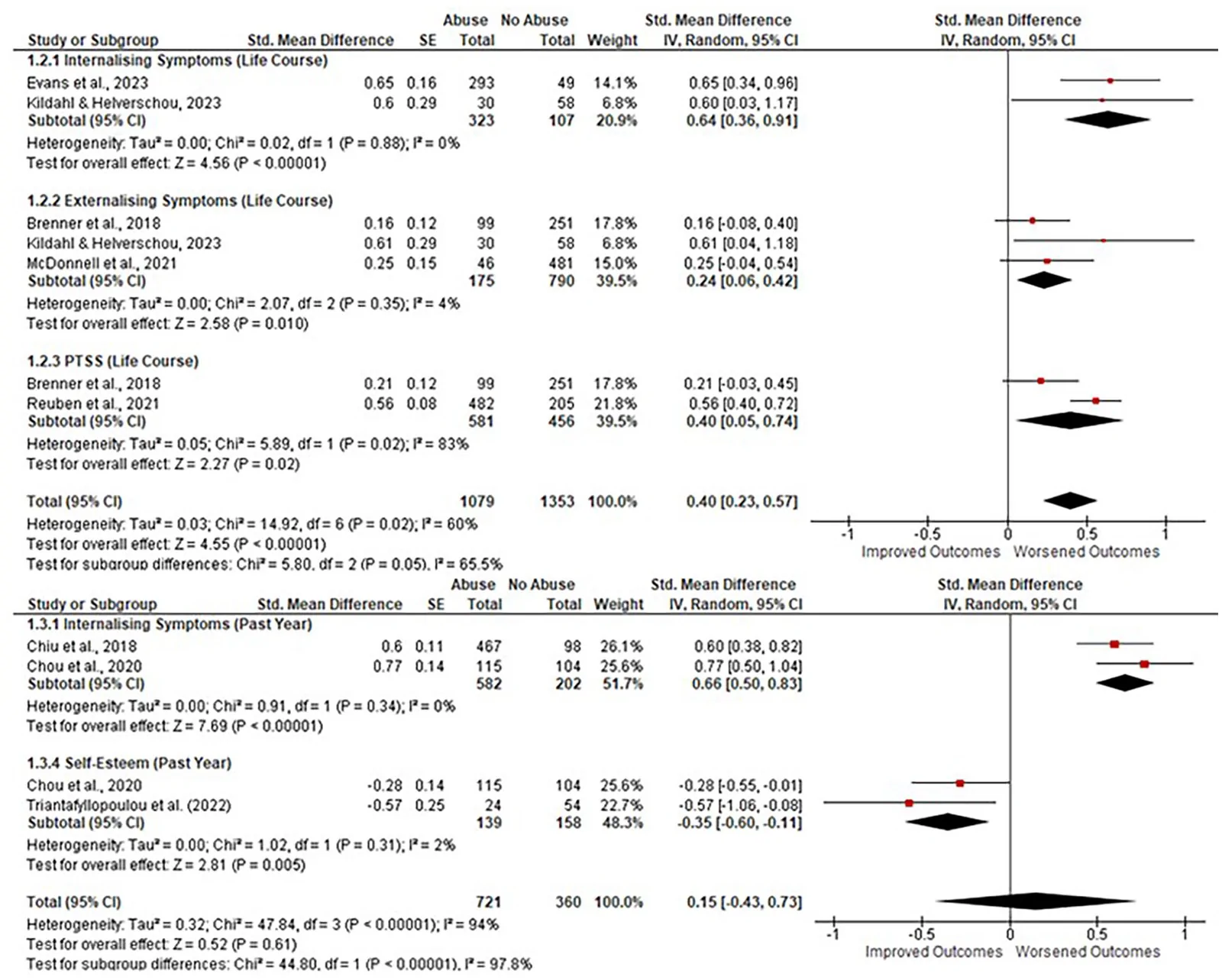
Forrest plot of associations between violence and mental health and behavioral factors: life course and past year.

Subgroup analyses found significant associations between interpersonal violence and all five outcomes examined (see Figure 2). The strongest association was a moderate-to-large correlation with greater internalizing symptoms, *d* = 0.66, *p* < .001; CI [0.51, 0.80]. This was followed by a moderate-to-large association with greater suicidal thoughts and behavior, *d* = 0.63, *p* < .001; [0.44, 0.82] and PTSS, *d* = 0.41, *p* < .05; [0.08, 0.73], a weak-to-moderate association with lower self-esteem, *d* = −0.35, *p* < .01;[−0.60, −0.11], and a weak-to-moderate association with increased externalizing behavioral challenges, *d* = 0.22, *p* < .01; [0.07, 0.37]. This trend also emerged in follow-up analyses of mental health and behavioral challenges across the life course and within the past year (see Figure 3).

### Homogeneity Analysis

We assessed homogeneity using chi-square (χ^2^) and found significant and high levels of heterogeneity for the PTSS (*I*^2^ = 81%, *p* < .05; *cf.* Higgins et al., 2003) subgroup analysis only. It is unclear why nonsignificant levels of heterogeneity were found for the four remaining subgroup analyses, particularly as other subgroup analyses had a small number of studies with substantial variance between them (e.g., participant characteristics and measurement/violence types), and this is a common factor from which heterogeneity derives (Gagnier et al., 2012; Liberati et al., 2009). However, as we have implemented comprehensive methods for study inclusion and the quantitative synthesis trends in an expected direction, we report these findings but urge caution when interpreting results given these considerations. The results of each analysis, including results relating to heterogeneity, are provided in Figures 2 and S1.

### Thematic Synthesis of Violence Outcomes

We conducted a thematic synthesis of the remaining 57 studies, comprising quantitative, mixed methods, and qualitative designs, not included in the meta-analysis due to use of qualitative methods, noncomparable quantitative measures, or outcomes that were not examined by two or more studies. These studies were predominantly cross-sectional (87.7%), with longitudinal findings reported where relevant. A condensed thematic synthesis of the most salient individual and social outcomes associated with experiences of violence among autistic people, organized by domain and life stage, is outlined below. Where reported, we have indicated distinctions between violence exposure likelihood or frequency, and for recent and lifetime violence experiences. A detailed review of all included findings pertaining to individual and social sequelae associated with autistic experiences of victimization by violence type is provided in Supplemental Information: Expanded Results.

The association between violence and individual-level outcomes was examined by 41 studies (26 quantitative, 9 qualitative, 6 mixed methods) comprising 33,806 participants (11,486 autistic and 22,320 non-autistic). Autistic people reporting the occurrence and recurrence of lifetime exposure to bullying or combined forms of violence (e.g., physical, sexual, and psychological) experienced worsened intra- (e.g., white matter disruption, more mental health conditions, internalizing symptoms, PTSS, and suicidality) and interpersonal difficulties (e.g., behavioral challenges, conduct issues, and impulsivity) from childhood compared to non-autistic counterparts (*n* = 5 studies). The presence and recurrence of recent and lifetime exposure to one (e.g., bullying, discrimination, and neighborhood violence) or a variety of combined violence types was most frequently found to be associated with immediate and long-lasting detrimental outcomes for mental health (*n* = 34 studies; e.g., negative self-concept, internalizing stressors or symptomology, PTSS, self-harm, suicidality), followed by behavior (7 studies; e.g., hyperactivity, noncompliance, retaliation, and conduct problems), and repeated victimization into adulthood (*n* = 2 studies). Retrospective, qualitative accounts from adulthood provided insight into how autistic people responded to the occurrence of lifetime exposure to intersecting violence types, including a tendency to desensitize these experiences (*n* = 2 studies) and respond to initial and recurring exposure by utilizing survival strategies (e.g., fawning, masking, and shutting down) informed by autism-related stigma learned at home, school, and/or in therapy (*n* = 5 studies). Such accounts also suggest that autistic people experience barriers to disclosing multiple forms of abuse experienced across the life course, including fear of not being believed or adverse consequences from not meeting others’ expectations (*n* = 2 studies). Moreover, those who did disclose lifetime violence exposure to combined victimization types experienced communication barriers (*n* = 2 studies), inadequate support or ostracization from family, friends, or partners (*n* = 3 studies), and/or negative encounters with community professionals (e.g., police and therapists), schools, and the criminal justice system (*n* = 4 studies).

The association between violence and social-level outcomes was examined by 20 studies (7 quantitative, 9 qualitative, 4 mixed methods) comprising 14,194 participants (6,014 autistic, 8,180 non-autistic). Most studies (*n* = 19) included autistic participants only. A recurring trend emerged whereby the presence and recurrence of recent and lifetime exposure to one (bullying and cyberbullying) or multitype violence exposure (e.g., physical, community, and sexual) was associated with psychological/emotional responses and adaptive or protective changes in social behavior (e.g., self-filtering, assertiveness, avoidance; *n* = 13 studies). These changes were, in turn, associated with diminished relational outcomes (*n* = 13 studies; e.g., reduced social confidence/prosocial behavior, unhealthy relationship dynamics, poor social well-being) and increased social vulnerability (*n* = 8 studies; e.g., isolated, ostracized, trapped/increased dependence) from early childhood into older adulthood. The relationship between the presence and recurrence of one (bullying, discrimination, and abuse in therapy) or multiple (sexual, verbal, and physical) lifetime violence types from childhood and social-related difficulties were observed in the home (*n* = 3 studies; e.g., destabilized routines, housing and financial instability, reinforced harmful therapy) and across educational and employment contexts (*n* = 7 studies; e.g., school or university refusal/drop-out, feeling singled-out or undermined for being denied accommodations in academic settings, being fired). Analyses of cross-sectional and longitudinal data also found a correlation between the presence and recurrence of physical, sexual, and psychological abuse from childhood and adolescence, involving greater service use (*n* = 2 studies), as well as and disruptions to normative development in understanding relationships and sexuality (*n* = 1 study; e.g., lack of education or open dialogue, reliance on pornography). Strategies identified as helpful in preventing or recovering from multiple lifetime violence experiences included early safety education (e.g., teaching boundaries, healthy relationships, and recognizing abusive behavior), fostering meaningful and respectful relationships, and having access to inclusive therapy and communities (*n* = 1 study).

Gender-related differences in the correlates of violence were explored by 9 studies (8 quantitative, 1 mixed methods) comprising 5,245 participants (3,864 autistic and 1,381 non-autistic). These differences were observed in the association between varying forms of violence and mental health symptoms across cross-sectional and longitudinal data from childhood. Among children and adolescents, quantitative analysis found that the occurrence of lifetime physical and emotional abuse was related to increased behavioral and parenting concerns among girls than boys (*n* = 1 study). Both quantitative and qualitative accounts indicated that the likelihood and frequency of recent and lifetime bullying related to greater internalizing symptoms (anxiety, depression, insecurity; *n* = 2 studies), self-blame (*n* = 1 study), and a higher risk of suicidal thoughts and behavior during a 5-year follow-up period (*n* = 1 study) among girls than boys. No clear gender-related differences emerged between the likelihood and frequency of lifetime bullying experiences and educational outcomes (*n* = 3 studies; e.g., school refusal; academic problems), nor for utilizing services for child abuse (*n* = 1 study). However, exposure to multiple abuse types in childhood and adolescence was associated with fewer diagnoses during emergency psychiatric care among autistic girls and boys compared to non-autistic boys and girls (*n* = 1 study). This suggests that gender alone may not relate to how victimization is expressed and identified among autistic populations in crisis assessments. In adulthood, a history of physical and sexual lifetime trauma was associated with an increased likelihood of meeting PTSD criteria among nonbinary people (65%), followed by transgender women (58%), transgender men (54%), women (42%), and men (33%; *n* = 1 study).

## Discussion

In this systematic review and meta-analysis, we examined the association between experiences of interpersonal violence and individual and social outcomes as they emerged across the life course. We also assessed how these difficulties might interact with gender to shape susceptibility toward victimization outcomes among autistic people of any age and support level. Results from our quantitative and qualitative syntheses found violence to be associated with a range of negative and enduring individual-level outcomes observable from childhood, including white matter disruption, mental health issues (e.g., anxiety, depression, PTSS, suicidal thoughts and behavior, and lower self-esteem), and behavioral concerns (e.g., conduct issues, noncompliance, and trauma-response strategies). This was supported by our thematic synthesis, whereby violence was associated with immediate and long-lasting negative outcomes individually (e.g., white matter disruption, more mental health conditions/symptoms) and behaviorally (e.g., behavioral/conduct challenges, impulsivity) compared to non-autistic controls from childhood. At a social level, violence was linked to worsened relational (e.g., perceived competence, relationship dynamics, and social well-being) and communal outcomes (e.g., worsened employment and educational outcomes, greater service use) across the lifespan. Gender-related trends emerged in mental health outcomes associated with victimization, with females reporting increased intra- (e.g., negative self-concept and mental health challenges) and interpersonal challenges (e.g., conflict management) from early childhood. Preliminary data also further suggest females and gender minorities may be more likely to experience PTSD following victimization in adulthood. Together, these trends lend support for minority stress and intersectional theories (Crenshaw, 1993; Grollman, 2014; Meyer, 2003). Findings from our review provide preliminary, yet comprehensive, insight into far-reaching and enduring harm, individually and contextually, associated with interpersonal violence experiences among autistic people throughout the life course. A summary of the most critical findings is listed in Table 2.

**Table 2.** Summary of Critical Findings.

Key Outcomes
*Meta-Analysis*: Experiencing one or more violence types was significantly associated with increased mental health and behavioral difficulties. The strongest association was for internalizing symptoms (e.g., depression, anxiety, insomnia; *d* = 0.66, *p* < .001; 95% CI [0.51, 0.80]), then suicidal thoughts and behavior (*d* = 0.63, *p* < .001; [0.44, 0.82]), post-traumatic stress symptoms (e.g., intrusion symptoms, avoidance, altered arousal/reactivity; *d* = 0.41, *p* < .05; [0.08, 0.73]), lower self-esteem (*d* = −0.35, *p* < .01; [−0.60, −0.11]), and externalized behavioral difficulties (e.g., opposition, hyperactivity/impulsivity, inattention; *d* = 0.22, *p* < .01; [0.07, 0.37]).
*Narrative Review*: Violence was associated with worsened individual neurocognitive and psychosocial sequelae across the life course among autistic people compared to non-autistic people. Individually, victimization from childhood related to greater white matter disruption, negative self-concept, mental health (e.g., internalizing stressors/symptomology, PTSS, self-harm, and suicidality) and behavioral challenges (e.g., irritability, retaliation, and conduct issues), and repeated victimization. These experiences tended to be internalized and responded to with survival strategies informed by autism-related stigma learned across communal contexts from early childhood. Autistic participants reported significant communication barriers, inadequate support, and negative interactions with community professionals when disclosing abuse.
Negative psychological/emotional and protective responses to violence are related to negative outcomes for relational development (e.g., social confidence, relationship dynamics, and social well-being) and heightened social vulnerability (e.g., lack of socio-sexual safety education, isolation, trapped, dependent). Lifelong associations across community contexts included destabilization in the home, increased service use (e.g., police contacts, emergency room visits, hospitalization), and disadvantage in employment and education settings.
Victimization was associated with greater difficulty both individually (e.g., negative self-concept, worsened mental health) and socially (e.g., parenting concerns and conflict management) among females, and greater PTSD among gender minorities, compared to males. However, autistic boys and girls were less likely to receive diagnoses in emergency psychiatric care for abuse than non-autistic boys and girls.
Key Implications of Review
*Practice:* Autism-specific training among frontline community professionals (e.g., teachers, clinicians, police), particularly those contacted by autistic people in crisis (e.g., doctors, nurses, police, and counselors).
Recognition among clinicians and healthcare workers of the potential ways autistic people may differentially identify, process, manage, and communicate violence (e.g., internalizing these experiences and masking difficulty, self-blame, greater risk of suicidal thoughts and behavior).
*Policy:* Funding should be invested in institutional safeguard mechanisms and response strategies for autistic people and their families at risk of or affected by violence, including, but not limited to: (a) best-practice responses that consider demographic diversity (e.g., age, gender, and culture); and (b) autism training among frontline community professionals, particularly crisis workers.
A strategic supply of culturally sensitive and accessible support provided to autistic people (of any age) and their families at risk of or impacted by violence, including: (a) financial and housing assistance; (b) adequate access to specialized mental health care; and (c) provision of additional, evidence-based support resources (e.g., safety information, counseling or community support initiatives, and crisis assistance).
*Research:* Research on the associated impact of violence across various individual and social contexts throughout development among autistic gender minorities and autistic people with higher support needs (e.g., co-occurring ID and communication difficulties).
Additional research on ways in which violence may be differentially identified, processed, managed, and communicated among autistic people, and best-practice response strategies in terms of crisis de-escalation and therapeutic approaches among autistic people and their families who are impacted by trauma.
Research that considers the relational dynamic between violence perpetrators and autistic people who are victimized to additional risk, including risk factors for offending.
Research on age-appropriate safety education initiatives aimed at providing optionally accessible, informative socio-sexual safety education that teaches boundaries, healthy relationship dynamics, and recognizing/responding to risks, etc.

*Note.* ID = intellectual disability; PTSD = post-traumatic stress disorder; PTSS = post-traumatic stress symptomology.

### Outcomes of Interpersonal Violence

Neurodevelopmental differences that characterize autism (Bird & Cook, 2013; Ecker et al., 2015; Zixuan et al., 2024) are associated with additional, stress-inducing psychosocial processes, including white matter disruption, negative self-concept, internalizing and externalizing difficulties, lack of social support, and repeated traumatization (e.g., Gibbs & Pellicano, 2023; Mandell et al., 2005; Yoshikawa et al., 2022). These processes appear to share a bidirectional relationship with violence, enhancing its risks and impacts from early development (Cooke et al., 2024; Kerns et al., 2015). Most notably, review findings suggest exposure to violence is associated with greater exacerbation of functional and structural neural differences between autistic and non-autistic children (Yoshikawa et al., 2022). This may contribute to an increased likelihood that autistic people will experience more pronounced and enduring difficulties with how interpersonal trauma is identified (e.g., difficulty interpreting socioemotional contexts), processed (e.g., differential emotional processing and dysregulation), managed (e.g., heightened response to perceived threats and overactive physical response), and communicated (e.g., difficulty verbalizing experiences; Cooke et al., 2024; Kerns et al., 2015, 2022; Lim et al., 2020; Pearson et al., 2023; Zixuan et al., 2024). This appears to involve a greater tendency toward internalizing violence and experiencing related symptomology, such as anxiety, depression, interpersonal sensitivity, insomnia, and suicidal thoughts and behavior, with more external behavioral issues appearing less likely (see Figure 2). This may arise from cumulative exposure to lifetime violence (e.g., Evans et al., 2023), compounded by autism-related stigma and barriers to identifying or supporting trauma disclosure (Anderson, 2023; Darazsdi & Bialka, 2023; Miller et al., 2022). In turn, suicidal thoughts and behavior may be utilized as a response mechanism to insufficiently addressed or treated internalized pain or distress. Moreover, PTSS may be under-identified due to diagnostic overshadowing, variability in measurement approaches, or under-reported trauma (see Peterson et al., 2019). Taken together, these findings have important implications for underlying processes, unique to autism, that enhance victimization risk and impacts (Cooke et al., 2024; Kerns et al., 2015). They also demonstrate the urgency of additional research on best-practice response strategies to prevent or mitigate the individual correlates of violence, including adequate access to specialized mental health care (Cooke et al., 2024; Cooper & Whittingham, 2022; Pearson et al., 2023).

Individual experiences of violence are not without social influence. Specifically, we suggest that intrapersonal features (e.g., mental health, autism characteristics, and trauma-related responses) interact with factors in the social environment (e.g., isolation, disparate power relationships, housing instability, and inadequate support services) to contribute to negative outcomes for relational functioning, personal safety, and service use following victimization from childhood. For example, review findings suggest that exposure to childhood violence is associated with experiencing other forms of victimization in the same year (Pfeffer, 2016), greater service use during childhood and adolescence, such as hospitalization, emergency room visits, or police contacts (Mandell et al., 2005; McDonnell et al., 2021), and further abuse as an adult (Douglas & Sedgewick, 2023). Moreover, violence destabilizes the childhood home (e.g., routines, reinforced harmful therapy, housing instability, and finances; Anderson, 2023; Kerns et al., 2022; Pearson et al., 2022) and contributes to mental health and behavioral concerns (e.g., Figure 2). Together, these factors appear to contribute to disrupted relational development (e.g., social competence, socio-sexual learning, relationship dynamics, and social well-being) and are associated with increased social vulnerability into adulthood, including isolation, ostracization, feeling trapped, or increased dependence (Douglas & Sedgewick, 2023; Gibbs & Pellicano, 2023; Heselton et al., 2022; Kerns et al., 2022; Leung et al., 2023; Pearson et al., 2022, 2023; Rothman & Graham Holmes, 2022). So considered, adequate structural support to families at risk of violence, such as financial, mental health, coping support, access to early safety education (e.g., boundaries, healthy relationship dynamics, and recognizing abuse or risks), and improved access to community support (Cooke et al., 2024; Douglas & Sedgewick, 2023; Hancock et al., 2017; Joyal et al., 2021; Pearson et al., 2023) could have far-reaching and long-lasting potential to improve healthy relationship development, functioning, safety, and community connectedness in autism following violence.

Autism and gender-related differences in how violence is internally processed and outwardly expressed appear to intersect with disparities in the provision of support, enhancing susceptibility toward enduring neurocognitive and psychosocial sequelae among females and gender minorities (e.g., Greenlee et al., 2020; Holden et al., 2020; McDonnell et al., 2021; Reuben et al., 2021; Sedgewick et al., 2019). Victimization is associated with enhanced challenges for girls in terms of formative development, particularly regarding conflict management (e.g., withdrawal and hopelessness) and negative self-concept (e.g., self-blame; Sedgewick et al., 2019), behavioral concerns (McDonnell et al., 2021), internalizing symptomology (Greenlee et al., 2020; Sedgewick et al., 2019), and suicidal thoughts and behavior (Holden et al., 2020). These patterns are broadly reflected in general population literature, whereby women and gender minorities experience elevated psychological harms (e.g., PTSD, depression, and suicidal thoughts and behavior) associated with multiple forms of violence exposure (Brown & Herman, 2015; Johns et al., 2019; Sardinha et al., 2022). However, these disparities appear to be compounded by autism-specific factors. To extend reasons for these disparities tendered by Cooke et al. (2024), we suggest that disparities are multifaceted, involving interaction between intrapersonal vulnerability (e.g., autism *and* gender marginalization) and inadequate institutional safeguard mechanisms aimed at mitigating violence risk and identifying and supporting autistic people affected by violence. For example, autistic people, most notably females, have greater stressor exposure and are more susceptible to the emotional impacts of relational stressors and violence (e.g., bullying, chronic entrapment, and verbal violence, neglect; Haruvi-Lamdan et al., 2020; McQuaid et al., 2022; Moseley et al., 2024; Zeedyk et al., 2014) yet tend to minimize, desensitize, and internalize these experiences (Gibbs & Pellicano, 2023; Pearson et al., 2023). It is possible this arises from, or is compounded by, harmful therapeutic practices that discourage self-trust, agency, and authentic expression during formative development (e.g., Anderson, 2023; Evans et al., 2023). This may also be due to increased expectations for females to display prosocial, socially adept, and compliant behavior (Dean et al., 2017; Maatta & Uusiautti, 2020; Martin & Beese, 2017; Mo et al., 2022), and/or autism- and/or gender-related barriers, stigma, or discrimination when seeking support (e.g., communication difficulties, ostracization, medical sexism; Anderson, 2023; Darazsdi & Bialka, 2023; Miller et al., 2022; Pearson et al., 2023). Importantly, our review raises significant concerns that autistic people are not only placed in positions where they are susceptible to further abuse from a young age, but appear conditioned to respond with social desirability (e.g., fawning), superficial adaptivity (e.g., masking), and dissociation (e.g., shutting down; Anderson, 2023; Douglas & Sedgewick, 2023; Evans et al., 2023; Fisher & Taylor, 2016; Mo et al., 2022; Pearson et al., 2023).

### Strengths and Limitations

In this review and analysis, we provide a comprehensive and contextual outline of violence experiences and their correlates for autistic people throughout development, both individually and socially. These findings demonstrate the utility of applying minority and intersectional models (Crenshaw, 1993; Grollman, 2014; Meyer, 2003) to improve our understanding of violence to improve mitigation efforts (Cooke et al., 2024; Pecora et al., 2016, 2021) and understand and address disparities in outcomes following violence among autistic people compared to the broader population. Importantly, the review of quantitative (Figure 2) and qualitative findings provide initial insight into how these associated outcomes may be differentially experienced within autism, and highlight a dire need for broader structural improvement in how community professionals, family members, and institutions recognize and support autistic people at risk of or affected by violence.

Our study is not without limitations. We conducted subgroup analyses on studies that examined violence outcomes in two or more studies; however, we note this small number limits statistical power and interpretive reliability. Larger datasets are needed for more robust and reliable subgroup analyses in future works. Participants included in our review were predominantly white (73%), while autistic participants were primarily male (71%) and without ID (56%) and including a substantially lower number of gender minorities (e.g., nonbinary, transgender; 0.5–1.9%) compared to autism gender variance estimates (4–16%; Kallitsounaki & Williams, 2023). Indeed, we found that a nonbinary approach to the collection of gender data only occurred as recently as 2019, significantly limiting our ability to test evidence for gender effects. There were limited studies included in this review that examined specific violence outcome differences for gender minorities, which primarily limited our qualitative examination to binary gender differences, while the relative number of studies (31%) that did not specify whether participants had co-occurring ID restricted our examination of its influence on violence outcomes. Despite the comprehensiveness of this review, it is critical to acknowledge that autistic people with greater communication challenges (e.g., non-speaking) are often under-represented in research, yet face increased vulnerability due to increased dependence and decreased access to support (e.g., Cooke et al., 2024). Consequently, these issues limited the generalisability of our findings to the broader autism population and warrant additional research in this area among these groups. Though we observed high levels of heterogeneity in our meta-analysis (93%), this can be accounted for by substantial variance in outcome and measurement tools and should be interpreted considering this. Further, the high number (80%) of cross-sectional studies in this review confines our ability to derive definitive causal conclusions from our findings and risks recall bias. While a small proportion of included studies had low quality (10%), they presented issues with statistics (e.g., missing data, unreliable statistical model, small sample size), selection bias, and measurement bias.

### Implications and Future Directions

The findings of this study highlight key areas for future practice and policy. Most notably, our findings extend the field by characterizing the complex and likely bidirectional relationship between internalizing/externalizing difficulties, which are often heightened in autism, and experiences of violence (Cooke et al., 2024; Kerns et al., 2015). This interplay appears to contribute to psychosocial processes that increase autistic vulnerability toward violence with lifelong, cascading, and multifaceted effects for intrapersonal and interpersonal health, functioning, development, connectedness, safety, and service. Our findings strengthen the call to improve institutional safeguard mechanisms and response strategies for autistic individuals and families at risk of or experiencing violence (Cooke et al., 2024). Priority initiatives include integrating evidentiary support for best-practice response strategies that consider differences in trauma identification and expression in autism as well as demographic diversity (e.g., differential outcomes or disparities in care, trauma-informed care, restorative practice; Cooper & Whittingham, 2022; Faccini & Allely, 2021; Shimmin et al., 2017). In addition, there is a need for autism-specific training among frontline community professionals (e.g., teachers, police, clinicians, support workers, child protection), and improved access to specialized mental health care, and practical support resources (e.g., safety information, financial and housing assistance, counseling services, and crisis assistance). Finally, fostering inclusive community support networks for autistic people throughout the life course has been identified as a salient violence prevention and response initiative (Cooke et al., 2024; Coorg & Tournay, 2012; Guan et al., 2021; Hwang et al., 2020; Pearson et al., 2023). See Table 3 for an outline of implications for practice, policy, and research.

**Table 3.** Key Implications of Review.

*Practice*: Autism-specific training among frontline community professionals (e.g., teachers, clinicians, and police), particularly those contacted by autistic people in crisis (e.g., doctors, nurses, police, counsellors, etc.).
Recognition among clinicians and healthcare workers of the potential ways autistic people may differentially identify, process, manage, and communicate violence (e.g., internalizing these experiences and masking difficulty, self-blame, greater risk of suicidal thoughts and behavior).
*Policy*: Funding should be invested in institutional safeguard mechanisms and response strategies for autistic people and their families at risk of or affected by violence, including, but not limited to: (a) best practice responses that consider demographic diversity (e.g., age, gender, and culture); and (b) autism training among community professionals, particularly crisis workers.
A strategic supply of culturally sensitive and accessible support provided to autistic people (of any age) and their families at risk of or impacted by violence, including: (a) financial and housing assistance; (b) adequate access to specialised mental health care; and (c) provision of additional, evidence-based support resources (e.g., safety information, counselling or community support initiatives, crisis assistance).
*Research*: Research on the associated impact of violence across various individual and social contexts throughout development among autistic gender minorities and autistic people with higher support needs (e.g., co-occurring ID, communication difficulties).
Additional research on ways in which violence may be differentially identified, processed, managed, and communicated among autistic people, and best practice response strategies in terms of crisis de-escalation and therapeutic approaches among autistic people and their families who are impacted by trauma.
Research that considers the relational dynamic between violence perpetrators and autistic people who are victimised to additional risk, including risk factors for offending.
Research on age-appropriate safety education initiatives aimed at providing optionally accessible, informative socio-sexual safety education that teaches boundaries, healthy relationship dynamics, and recognizing/responding to risks, etc.

## Conclusion

Reviewed findings strongly suggest exposure to interpersonal violence is associated with long-lasting and far-reaching harm among autistic people relative to the broader population, highlighting a potential enhanced and enduring susceptibility toward violence sequelae among females and gender minorities. Our analysis found violence was related to a range of mental health and behavioral challenges, with the strongest associations found for internalizing symptomology and suicidal thoughts and behavior. This was supported and expanded by our thematic synthesis, which indicated that violence is linked to intra- and interpersonal difficulties, which often co-occur with autism. Findings also point to a failure of safeguard and response mechanisms aimed at identifying and responding to autistic people at risk of or impacted by violence. This was most indicated among females from formative development. Our review emphasizes the need for initiatives aimed at integrating best-practice response strategies with adequate autism training among community professionals and access to specialized health care, support services, and community opportunities among autistic people throughout the life course.

## Supplemental Material

sj-docx-1-tva-10.1177_15248380251357618 – Supplemental material for Outcomes of Experiencing Interpersonal Violence in Autism: A Mixed Methods Systematic Review and Meta-Analysis Supplemental material, sj-docx-1-tva-10.1177_15248380251357618 for Outcomes of Experiencing Interpersonal Violence in Autism: A Mixed Methods Systematic Review and Meta-Analysis by Kassandrah Cooke, Kathryn Ridgway, Laura Pecora, Elizabeth Westrupp, Darren Hedley, Merrilyn Hooley and Mark A. Stokes in Trauma, Violence, & Abuse

## Notes

1. Identity-first language will be used throughout to reflect the preferences of people diagnosed with autism spectrum disorder (e.g., Bury et al., 2020; Keating et al., 2022). The term “autistic” will be used throughout.
