# Supplementary material for: Outcomes of Experiencing Interpersonal Violence in Autism: A Mixed Methods Systematic Review and Meta-Analysis
Source: Trauma Violence Abuse. 2025 Aug 9;27(4):1584–601. doi: 10.1177/15248380251357618 (PMC13578663; doi:10.1177/15248380251357618)
Supplement: Supplementary file 1 [file sj-docx-1-tva-10.1177_15248380251357618.docx]

**Supplementary Information**

**Expanded Methods: Study Selection and Data Extraction**

Following initial screening, duplicates were removed, and remaining titles and abstracts were screened by the first and second authors. Full-text screening was then conducted, followed by screening included studies reference lists to identify additional articles of relevance. The first author extracted coded data on study country, a description of the sample (e.g., co-occurring intellectual disability, gender, age), diagnostic details, data collection methods, outcome(s) examined, outcomes/findings, effect size (for studies that were included in the meta-analysis), reasons for exclusion from meta-analysis, and study quality.

**Table S1**

*Full Search Strategy for Included Studies: Autistic Experiences of Interpersonal Violence, Compared by Gender*

| Outcome |  | Descriptor |
| --- | --- | --- |
| Search for autism | 1. | “Autism Spectrum Disorder*” |
|  | 2. | “Pervasive Developmental Disorder?Not Otherwise Specified” |
|  | 3. | Autis* |
|  | 4. | ASD |
|  | 5. | ASC |
|  | 6. | PDD?NOS |
|  | 7. | Asperger* |
|  | 8. | OR 1-7 |
| Search for interpersonal violence | 9. | Violen* |
|  | 10. | Abus* |
|  | 11. | Assault* |
|  | 12. | ((Negative OR Adverse) N5 (Experience* OR Encounter*)) |
|  | 13. | Victim* |
|  | 14. | Stalk* |
|  | 15. | Rape* |
|  | 16. | Crim* |
|  | 17. | (Sex* N3 Offen?e) |
|  | 18. | Trauma* |
|  | 19. | Harrass* |
|  | 20. | Threat* |
|  | 21. | Coerc* |
|  | 22. | Intimidat* |
|  | 23. | Bull* |
|  | 24. | Cyber?bull* |
|  | 25. | Forc* |
|  | 26. | Manipulat* |
|  | 27. | Gaslight* |
|  | 28. | “Hate Speech” |
|  | 29. | OR 9-28 |
| Search for gender | 30. | Gender |
|  | 31. | ((Sex OR Gender) N5 (Difference* OR Based)) |
|  | 32. | (Male* N3 Female*) |
|  | 33. | (Men N3 Women) |
|  | 34. | (boy* N3 girl*) |
|  | 35. | OR 30-34 |
| Search for combinations | 36. | 8 AND 29 AND 35 |

**Table S2**

*Study Quality Assessment Using the Mixed-Methods Appraisal Tool (MMAT)**

| **Study** | **S1** | **S2** | **1.1** | **1.2** | **1.3** | **1.4** | **1.5** | **4.1** | **4.2** | **4.3** | **4.4** | **4.5** | **5.1** | **5.2** | **5.3** | **5.4** | **5.5** |
| --- | --- | --- | --- | --- | --- | --- | --- | --- | --- | --- | --- | --- | --- | --- | --- | --- | --- |
|  |  |  | **Qualitative** | | | | | **Quantitative descriptive** | | | | | **Mixed-methods** | | | | |
| Adams et al. (2016) | Y | Y | – | – | – | – | – | *x* | N | *x* | Y | Y | – | – | – | – | – |
| Anderson (2023) | Y | Y | Y | Y | Y | Y | Y | – | – | – | – | – | – | – | – | – | – |
| Andrzejewski et al. (2022) | Y | Y | – | – | – | – | – | Y | N | Y | Y | Y | – | – | – | – | – |
| Bemmouna et al. (2023) | Y | Y | – | – | – | – | – | Y | N | Y | Y | *x* | – | – | – | – | – |
| Botha et al. (2019) | Y | Y | Y | Y | Y | Y | Y | – | – | – | – | – | – | – | – | – | – |
| Botha & Frost (2020) | Y | Y | – | – | – | – | – | Y | *x* | Y | Y | Y | – | – | – | – | – |
| Brenner et al. (2018) | Y | Y | – | – | – | – | – | Y | Y | N | Y | Y | – | – | – | – | – |
| Bross et al. (2022) | Y | Y | Y | *x* | Y | Y | Y | Y | N | Y | Y | *x* | Y | Y | Y | Y | Y |
| Cappadocia et al. (2012) | Y | Y | – | – | – | – | – | Y | *x* | Y | Y | Y | – | – | – | – | – |
| Chiu et al. (2018) | Y | Y | – | – | – | – | – | Y | N | Y | Y | Y | – | – | – | – | – |
| Chou et al. (2020) | Y | Y | – | – | – | – | – | Y | N | Y | Y | Y | – | – | – | – | – |
| Courreges et al. (2023) | Y | Y | – | – | – | – | – | *x* | N | *x* | Y | Y | – | – | – | – | – |
| Dababnah et al. (2022) | Y | Y | Y | Y | Y | Y | Y | *x* | N | Y | N | Y | Y | Y | Y | Y | Y |
| Darazsdi & Bialka (2023) | Y | Y | Y | Y | Y | Y | Y | – | – | – | – | – | – | – | – | – | – |
| Davis et al. (2022) | Y | *x* | – | – | – | – | – | *x* | N | *x* | Y | Y | – | – | – | – | – |
| Douglas & Sedgewick (2023) | Y | Y | Y | Y | Y | Y | Y | – | – | – | – | – | – | – | – | – | – |
| Evans et al. (2023) | Y | Y | – | – | – | – | – | Y | N | Y | Y | Y | – | – | – | – | – |
| Ferrigno et al. (2022) | Y | Y | – | – | – | – | – | Y | *x* | *x* | Y | Y | – | – | – | – | – |
| Fink et al. (2018) | Y | Y | – | – | – | – | – | *x* | N | *x* | Y | Y | – | – | – | – | – |
| Fisher & Taylor (2016) | Y | Y | Y | *x* | Y | Y | Y | *–* | *–* | *–* | *–* | *–* | *–* | *–* | *–* | *–* | *–* |
| Gibbs & PellicaN (2023) | Y | Y | Y | Y | Y | Y | Y | *–* | *–* | *–* | *–* | *–* | *–* | *–* | *–* | *–* | *–* |
| Greenlee et al. (2020) | Y | Y | *–* | *–* | *–* | *–* | *–* | Y | N | *x* | Y | Y | *–* | *–* | *–* | *–* | *–* |
| Haruvi-Lamdan et al. (2020) | Y | Y | *–* | *–* | *–* | *–* | *–* | Y | N | Y | Y | Y | *–* | *–* | *–* | *–* | *–* |
| Heselton et al. (2022) | Y | Y | Y | Y | Y | Y | Y | *–* | *–* | *–* | *–* | *–* | *–* | *–* | *–* | *–* | *–* |
| Holden et al. (2020) | Y | Y | *–* | *–* | *–* | *–* | *–* | Y | Y | *x* | Y | Y | *–* | *–* | *–* | *–* | *–* |
| Hong et al. (2016) | Y | Y | *–* | *–* | *–* | *–* | *–* | Y | *x* | Y | Y | Y | *–* | *–* | *–* | *–* | *–* |
| Hu et al. (2019) | Y | Y | *–* | *–* | *–* | *–* | *–* | Y | N | Y | Y | Y | *–* | *–* | *–* | *–* | *–* |
| Kerns et al. (2022) | Y | Y | Y | *x* | Y | Y | Y | Y | N | *x* | Y | Y | Y | Y | Y | Y | Y |
| Kidahl & Helverschou (2023) | Y | Y | *–* | *–* | *–* | *–* | *–* | Y | N | *x* | Y | Y | *–* | *–* | *–* | *–* | *–* |
| Leung et al. (2023) | Y | Y | Y | *x* | Y | Y | Y | *–* | *–* | *–* | *–* | *–* | *–* | *–* | *–* | *–* | *–* |
| Lin et al. (2020) | Y | Y | *–* | *–* | *–* | *–* | *–* | Y | N | Y | Y | Y | *–* | *–* | *–* | *–* | *–* |
| Liu et al. (2022) | Y | Y | *–* | *–* | *–* | *–* | *–* | Y | N | *x* | Y | Y | *–* | *–* | *–* | *–* | *–* |
| Lu et al. (2022) | Y | Y | *–* | *–* | *–* | *–* | *–* | Y | N | Y | Y | *x* | *–* | *–* | *–* | *–* | *–* |
| Mandell et al. (2005) | Y | Y | *–* | *–* | *–* | *–* | *–* | Y | *x* | *x* | Y | Y | *–* | *–* | *–* | *–* | *–* |
| Marion et al. (2023) | Y | Y | Y | Y | Y | Y | Y | *x* | N | Y | Y | Y | Y | Y | Y | N | Y |
| MaY et al. (2013) | Y | Y | *–* | *–* | *–* | *–* | *–* | *x* | *x* | *x* | Y | Y | *–* | *–* | *–* | *–* | *–* |
| McClemont et al. (2020) | Y | Y | *–* | *–* | *–* | *–* | *–* | Y | N | *x* | Y | Y | *–* | *–* | *–* | *–* | *–* |
| McDonnell et al. (2021) | Y | Y | *–* | *–* | *–* | *–* | *–* | N | N | *x* | Y | Y | *–* | *–* | *–* | *–* | *–* |
| Miller et al. (2022) | Y | Y | *x* | *x* | Y | Y | Y | Y | N | Y | *x* | Y | N | Y | Y | Y | Y |
| Ochi et al. (2020) | Y | Y | *–* | *–* | *–* | *–* | *–* | Y | *x* | *x* | Y | Y | *–* | *–* | *–* | *–* | *–* |
| Pearson et al. (2022) | Y | Y | *x* | *x* | Y | Y | Y | *–* | *–* | *–* | *–* | *–* | *–* | *–* | *–* | *–* | *–* |
| Pearson et al. (2023) | Y | Y | Y | Y | Y | Y | Y | *–* | *–* | *–* | *–* | *–* | *–* | *–* | *–* | *–* | *–* |
| Pfeffer (2016) | Y | Y | Y | *x* | Y | Y | Y | *–* | *–* | *–* | *–* | *–* | *–* | *–* | *–* | *–* | *–* |
| Reuben et al. (2021) | Y | Y | *–* | *–* | *–* | *–* | *–* | Y | N | Y | Y | Y | *–* | *–* | *–* | *–* | *–* |
| Rodriguez et al. (2021) | Y | Y | *–* | *–* | *–* | *–* | *–* | Y | N | *x* | Y | Y | *–* | *–* | *–* | *–* | *–* |
| Rothman & Graham Holmes (2022) | Y | Y | Y | Y | Y | Y | Y | *–* | *–* | *–* | *–* | *–* | *–* | *–* | *–* | *–* | *–* |
| Sedgewick et al. (2019) | Y | Y | Y | Y | Y | Y | Y | Y | N | Y | Y | Y | Y | Y | Y | Y | Y |
| So et al. (2021) | Y | Y | *–* | *–* | *–* | *–* | *–* | Y | *x* | *x* | Y | Y | *–* | *–* | *–* | *–* | *–* |
| Tan et al. (2023) | Y | Y | Y | *x* | Y | Y | Y | *–* | *–* | *–* | *–* | *–* | *–* | *–* | *–* | *–* | *–* |
| Toseeb et al. (2018) | Y | Y | *–* | *–* | *–* | *–* | *–* | Y | Y | *x* | Y | Y | *–* | *–* | *–* | *–* | *–* |
| Toseeb et al. (2020) | Y | Y | *–* | *–* | *–* | *–* | *–* | Y | Y | *x* | Y | Y | *–* | *–* | *–* | *–* | *–* |
| Triantafyllopoulou et al. (2022) | Y | Y | *–* | *–* | *–* | *–* | *–* | *x* | N | Y | Y | Y | *–* | *–* | *–* | *–* | *–* |
| Ung et al. (2016) | Y | Y | *–* | *–* | *–* | *–* | *–* | *x* | N | Y | Y | Y | *–* | *–* | *–* | *–* | *–* |
| Weiss et al. (2015) | Y | Y | *–* | *–* | *–* | *–* | *–* | Y | *x* | *x* | Y | Y | *–* | *–* | *–* | *–* | *–* |
| Wright (2018) | Y | Y | *–* | *–* | *–* | *–* | *–* | Y | N | Y | Y | Y | *–* | *–* | *–* | *–* | *–* |
| Yoshikawa et al. (2022) | Y | Y | *–* | *–* | *–* | *–* | *–* | Y | N | Y | Y | Y | *–* | *–* | *–* | *–* | *–* |
| Zablotsky et al. (2013) | Y | Y | *–* | *–* | *–* | *–* | *–* | *x* | *x* | Y | Y | Y | *–* | *–* | *–* | *–* | *–* |
| Zablotsky et al. (2014) | Y | Y | *–* | *–* | *–* | *–* | *–* | *x* | *x* | Y | Y | Y | *–* | *–* | *–* | *–* | *–* |
| Zeedyk et al. (2014) | Y | Y | *–* | *–* | *–* | *–* | *–* | Y | N | Y | Y | Y | *–* | *–* | *–* | *–* | *–* |

Note: Y = Yes; N = No; *x* =unclear; *– =* not applicable; * Column Headings taken from the MMAT as follows:

**“Screening Questions (For All Types)**

S1. Are there clear research questions?

S2. Do the collected data allow to address the research question?

**1. Qualitative**

1.1. Is the qualitative approach appropriate to answer the research question?

1.2. Are the qualitative data collection methods adequate to address the research question?

1.3. Are the findings adequately derived from the data?

1.4. Is the interpretation of results sufficiently substantiated by data?

1.5. Is there coherence between qualitative data sources, collection, analysis, and interpretation?

**4. Quantitative Descriptive**

4.1. Is the sampling strategy relevant to address the research question?

4.2. Is the sample representative of the target population?

4.3. Are the measurements appropriate?

4.4. Is the risk of nonresponse bias low?

4.5. Is the statistical analysis appropriate to answer the research question?

**5. Mixed-Methods**

5.1. Is there an adequate rationale for using a mixed methods design to address the research question?

5.2. Are the different components of the study effectively integrated to answer the research question?

5.3. Are the outputs of the integration of qualitative and quantitative components adequately addressed?

5.4. Are divergences and inconsistencies between quantitative and qualitative results adequately addressed?

5.5. Do the different components of the study adhere to the quality criteria of each tradition of the methods involved?

***Figure S1***

*Funnel Plot of Heterogeneity for Association Between Violence and Mental Health and Behavioural Factors*

**Expanded Results: Violence Outcomes**

***Physical, Sexual, and/or Psychological Violence***

Twenty studies examined associations between combined forms of interpersonal violence and various individual and social outcomes across the life course. At an individual-level, violence was associated with adverse effects on mental health and was described as emotionally impactful, involving feelings of shame, confusion, and anger, and taking time to process (Pearson et al., 2023). Negative social events were reported as particularly distressing among autistic, compared to non-autistic adults, relating to increases in hyper-arousal post-traumatic stress symptomology (PTSS) among autistic, but not non-autistic adults, particularly females (Haruvi-Lamdan et al., 2020). Experiencing one or more forms of violence related to mental health challenges such as: 1) increased internalising symptoms (e.g., anxiety, depression, lethargy; Brenner et al., 2018; Kildahl & Helverschou, 2023; Liu et al., 2022); 2) post-traumatic stress disorder (PTSD; Douglas & Sedgewick, 2023) and symptomology ([PTSS]; e.g., dissociative symptoms, irritability, agitation; Haruvi-Lamdan et al., 2020; Kildahl & Helverschou, 2023; Reuben et al., 2021), and; 3) suicidal thoughts and behaviour (Liu et al., 2022). Moreover, autistic females, transgender men, and non-binary people had an increased likelihood of meeting criteria for PTSD following interpersonal trauma compared to autistic men and transgender women, with non-binary people observed as particularly susceptible (Reuben et al., 2021).

Responses to violence primarily involved survival strategies such as shutting down, people pleasing or fawning (e.g., ignoring own needs to serve others), and masking autism characteristics (Anderson, 2023; Douglas & Sedgewick, 2023; Evans et al., 2023; Pearson et al., 2023). This derived from autistic participants being taught at home, in therapeutic contexts, or at school their natural response is “wrong” (Anderson, 2023; Douglas & Sedgewick, 2023), and being fearful of not meeting others' expectations (Pearson et al., 2023). These responses were exhausting (leading to burnout) and resulted in embarrassment, demoralisation, isolation, and challenges with identity denial (Pearson et al., 2023). Other responses included more externalised behaviours, including hyperactivity or non-compliance (Kildahl & Helverschou, 2023). Autistic people tended to be reluctant to disclose violence experiences due to fear of not being believed or getting in trouble, and difficulty communicating what happened (Douglas & Sedgewick, 2023; Pearson et al., 2023). Autistic people who did disclose violence experiences reported being discredited, disbelieved, or blamed (Douglas & Sedgewick, 2023; Pearson et al., 2023).

Autistic people described violence as emotionally impactful, but minimised these experiences, viewing them as inevitable, blaming themselves (Douglas & Sedgewick, 2023). Experiencing violence also involved negative feelings (e.g., anger, shame, confusion) and self-perceptions, including a lack of agency, difficulty trusting self- or self-judgement, feeling naïve or too trusting (Heselton et al., 2022; Pearson et al., 2022), and lower self-esteem (Liu et al., 2022). Participants also reported fear of being taken advantage of, difficulty trusting others, particularly authority figures, and expecting further abuse (Pearson et al., 2022, 2023). Learning autistic identity assisted with self-acceptance and making sense of victimisation experiences (Pearson et al., 2023).

At a proximal social level, the impact of violence was reported to be underestimated due to being autistic, including not being believed, blamed, or losing/being denied support or protection from adults, peers, family, or intimate partners (Douglas & Sedgewick, 2023; Kerns et al., 2022). Among autistic adults, already limited social support was reported to be worsened through intimate partners' abusive tactics (e.g., gaslighting, discrediting) and feeling trapped due to inadequate support, making it harder to leave and increasing social isolation and dependency on partner (Douglas & Sedgewick, 2023; Pearson et al., 2023). A combination of these factors (i.e., abusive tactics and lack of support when disclosing abuse) had negative impacts on participants' ability to recognise and seek help with abusive behaviour and manage trauma (Douglas & Sedgewick, 2023).

At a community-level, autistic participants recalled varying experiences of violence across home and therapeutic contexts which incurred negative impacts from childhood. In one study, participants recalled upsetting feelings and negative memories of Applied Behaviour Analysis (ABA) from childhood relating to abuse of power/physical force, reduced agency, and cognition being mischaracterised, resulting in these practices being reinforced in the home (Anderson, 2023). Experiencing domestic violence resulted in difficulty maintaining one’s routine, housing instability and homelessness, and further trauma (Kerns et al., 2022). Autistic males and females were also less likely to receive a diagnosis for abuse than non-autistic males (*d* = -0.58) and females (*d* = -0.63), respectively, during emergency psychiatric care (So et al., 2021). When seeking help with or justice for violence experiences, autistic participants reported negative perceptions of and interactions with therapists, police, other professionals, and the criminal justice system ([CJS]; Miller et al., 2022; Pearson et al., 2023). This included being scared of the police due to excessive use of force and maltreatment, feeling the CJS was unhelpful, ineffective, and/or has an inadequate understanding of autism, as well as no action or follow-up being taken by the police (Miller et al., 2022).

At a broader-social level, early strategies aimed at teaching boundaries, healthy relationships, and recognising abusive behaviour were identified as useful in preventing victimisation (Pearson et al., 2023). Meaningful, accepting, and respectful relationships, accessible and inclusive therapists knowledgeable in autism, and connection to autistic and intersectional communities assisted with recovering from victimisation (Pearson et al., 2023).

Overall experiences of interpersonal violence were associated with a range of negative outcomes across the life course. Exposure to childhood violence was associated with experiencing other forms of victimisation in the same year (Pfeffer, 2016), disruptions to socio-sexual learning (e.g., no open dialogue about sex/relationships, no socio-sexual education, reliance on pornography), greater white matter microstructural disruption among autistic, compared to non-autistic people (Yoshikawa et al., 2022), and experiencing further abuse as an adult (e.g., normalising abuse/abusive relationships; Douglas & Sedgewick, 2023). Childhood violence exposure also impacted development and were associated with higher internalising (e.g., depression, anxiety), externalising challenges (e.g., anger, resentment, hyperactivity, conduct issues) among autistic (Andrzejewski et al., 2022; Heselton et al., 2022), but not non-autistic people (Andrzejewski et al., 2022). Severe ACEs resulted in lasting trauma (Dababnah et al., 2021), while repeated violence was desensitised, normalised (Gibbs & Pellicano, 2023; Pearson et al., 2023), and internalised as deserved due to being autistic (Pearson et al., 2023). This presented immediate and long-lasting adverse impacts on mental health (e.g., anxiety, stress, depression), self-concept (e.g., self-blame, low-self-worth, expecting dehumanising treatment), relationships, and finances (Gibbs & Pellicano, 2023; Pearson et al., 2022, 2023). These difficulties continued from childhood to adulthood and further impacted social functioning (e.g., making safer choices, feeling scared and suspicious of all potential partners, and being reserved and transactional with intimate partners; Douglas & Sedgewick, 2023; Heselton et al., 2022).

***Physical Violence***

Outcomes of physical violence were examined by two studies. Exposure to physical child abuse in childhood was associated with an increased likelihood of behavioural concerns (e.g., acting out, assaultive behaviour), running away from home, attempting suicide, and having academic problems (Mandell et al., 2005). Child physical abuse was also associated with greater child service use, including psychiatric hospitalisation, ER visits, and police contacts (McDonnell et al., 2021). Service use did not differ by gender (McDonnell et al., 2021).

***Sexual Violence***

Three studies examined outcomes of sexual violence. Sexual violence among autistic children and adolescents was associated with greater psychological distress (*d* = 0.34; Lu et al., 2022), suicide-related problems and attempts, self-injurious behaviour, challenging behaviour (e.g., irritability, agitation), and sexually acting out or assaultive behaviour. Associations were also found between sexual violence and running away from home, and having had prior psychiatric hospitalisations (Mandell et al., 2005), but not hyperactivity or non-compliance (Kildahl & Helverschou, 2023).

***Psychological Violence***

Eleven studies examined outcomes of various forms of psychological violence (e.g., exclusion, verbal abuse, stigma, harassment, neglect). These were associated with internalising symptoms (e.g., psychological distress, depression, anxiety; Anderson, 2023; Lin et al., 2020; Lu et al., 2022), internalised stigma (e.g., self-loathing, embarrassment, shame; Anderson, 2023; Darazsdi & Bialka, 2023), trauma-related symptomology (e.g., PTSS, perceived stress, panic attacks; Anderson, 2023; Davis et al., 2022), badly diminished self-esteem (Darazsdi & Bialka, 2023), and suicidal thoughts and behaviour (Lin et al., 2020). Experiencing autism-related stigma and stereotypes (e.g., discrimination, infantilisation) was described as particularly limiting and destructive, resulting in feeling dehumanised, disempowered and insecure, and having internalised stressors (e.g., internalised stigma, second guessing self/situations, expectations of rejection; Darazsdi & Bialka, 2023; Marion et al., 2023). This was reported as complex to manage (e.g., autism concealment, strategic disclosure, reclaiming language and identity, reframing autism; Botha et al., 2019; Marion et al., 2023) and adversely impacted self-esteem (Darazsdi & Bialka, 2023; Marion et al., 2023), social self-efficacy and satisfaction (Marion et al., 2023), and wellbeing (Botha & Frost, 2020).

At a community-level, being emotionally abused, dehumanised, and having agency reduced by caregivers (e.g., parents, therapeutic staff, teachers) during childhood and adolescence was associated with greater service use (e.g., psychiatric hospitalisation, police contacts, ER visits), greater parenting stress among girls than boys (McDonnell et al., 2021), and was associated with being accepting of external influence and long-lasting trauma into adulthood (Anderson, 2023; Evans et al., 2023; Kerns et al., 2022). Communication challenges presented issues with disclosing abuse & seeking support from child protective services (CPS), incurring additional trauma (Kerns et al., 2022). Further, autism-related stigma, stereotyped, and sexist beliefs resulted in discrimination across medical (e.g., delayed autism diagnosis due to being dismissed as a hysterical and anxious female; Darazsdi & Bialka, 2023), employment, academic contexts (e.g., undermined credibility/capability, denigrated, fired, characterised as incapable) for needing accommodations (Marion et al., 2023; Tan et al., 2023). This led to negative self-perceptions (e.g., feeling demotivated, sidelined, dismissed, excluded) and had adverse impacts academically (e.g., diminished professional relationships, university withdrawal; Marion et al., 2023; Tan et al., 2023).

***Bullying***

Bullying (e.g., emotional, physical, psychological) was described as incessant and inescapable (Kerns et al., 2022), resulting in physical injury (Zablotsky et al., 2013), with emotional responses ranging from minimising or normalising (Fisher & Taylor, 2016). The emotional impact of bullying was met with a heightened response (Kerns et al., 2022) and was worsened among autistic people (76%) compared to non-autistic controls (47%; *d* = 0.67; Zeedyk et al., 2014). Bullying was not associated with emotional dysregulation among autistic and non autistic participants (Bemmouna et al., 2023); however, it was associated with greater externalising challenges (e.g., conduct, hyperactivity, retaliation, emotional outburst; Cappadocia et al., 2012; Fink et al., 2018; Fisher & Taylor, 2016; Toseeb et al., 2018; Zablotsky et al., 2013) and a desire for immediate or future retaliation (Fisher & Taylor, 2016), worsened mental health within autism (e.g., more co-occurring mental health conditions; internalising symptoms, self-harm, suicidal thoughts and behaviour, emotional trauma; Bross et al., 2022; Cappadocia et al., 2012; Ferrigno et al., 2022; Fink et al., 2018; Heselton et al., 2022; Holden et al., 2020; Hu et al., 2019; Lu et al., 2022; Mayes et al., 2013; Rodriguez et al., 2021; Toseeb et al., 2018, 2020; Ung et al., 2016; Weiss et al., 2015; Wright et al., 2018; Zablotsky et al., 2013), fear of safety (Zablotsky et al., 2013), fear of being manipulated, rejected, or experiencing further abuse (Rothman & Graham Holmes, 2022), and reduced quality of life (Hong et al., 2016). Further, being teased for autistic traits was linked to more autistic masking behaviours (*d* = 0.66; Evans et al., 2023), while bullying did not moderate the relationship between school type and anxiety and depression symptoms during the pandemic (Courreges et al., 2023).

The impact of bullying differed by gender within autism, with females managing conflict differently to males (Sedgewick et al., 2019), and had increased internalising symptoms (e.g., anxiety, depression, insecurity), blamed themselves for the conflict or felt conflict was unresolvable (Greenlee et al., 2020; Sedgewick et al., 2019), and had greater suicidality (Holden et al., 2020). Males were less emotionally impacted by bullying conflict, viewing it as something that could be overcome (Sedgewick et al., 2019). There were no gender differences in overall educational outcomes among bullied participants (Adams et al., 2016); however, findings were mixed as to whether females were more likely to exhibit school refusal than males (McClemont et al., 2020; Ochi et al., 2020).

At a proximal social-level, bullying was associated with peer problems, fewer prosocial skills (Fink et al., 2018; Toseeb et al., 2020), and had detrimental effects on friendship quality (*d* = 0.67; Lu et al., 2022), including avoiding forming and maintaining friendships due to fear and prior bullying experiences (Rothman & Graham Holmes, 2022). Autistic females had fewer, more difficult-to-manage, friendships, and reduced social support than non-autistic girls, which may have compounded the emotional impacts of bullying (Sedgewick et al., 2019).

At a community-level, being bullied was associated with negative educational outcomes (Adams et al., 2016), including a negative classroom environment (*d* = 0.52; Lu et al., 2022), school refusal (35%; McClemont et al., 2020), school drop-out (Miller et al., 2022), less assistance with difficulties at school (*d* = 0.37; Lu et al., 2022), and feeling the school isn’t supportive of bullied students (Fisher & Taylor, 2016).

A range of long-term impacts of bullying across the life course included a higher risk of suicidality at least five years following victimisation, particularly among autistic females and autistic people without co-occurring ID (Holden et al., 2020). Being bullied was also associated with worsened social disconnection and exclusion (e.g., isolation, being less trusting, avoidance, ostracization, stigmatisation), which started in childhood and continued to adulthood (Fisher & Taylor, 2016; Kerns et al., 2022).

***Cyberbullying***

Being cyberbullied was associated with mood fluctuations, rumination, and significant stress (Leung et al., 2023), as well as lower self-esteem, particularly for personal information being posted online (*d* = -0.52) and being excluded or ignored by others (*d* = - 0.61; Triantafyllopoulou et al., 2022). Relationally, cyberbullying was also associated with being ostracised, while unwanted sexual behaviours from someone met online resulted in mistrust and avoidance of future relationships (Leung et al., 2023).

**References**

*Anderson, L. K. (2023). Autistic experiences of applied behavior analysis. *Autism*. https://doi:10.1177/13623613221118216

*Andrzejewski, T., DeLucia, E. A., Semones, O., Khan, S., & McDonnell, C. G. (2022). Adverse Childhood Experiences in Autistic Children and Their Caregivers: Examining Intergenerational Continuity. *Journal of Autism and Developmental Disorders*, *53*, 2663-2679. https://doi.org/10.1007/s10803-022-05551-w

*Bemmouna, D., Lagzouli, A., & Weiner, L. (2023). The biosocial correlates and predictors of emotion dysregulation in autistic adults compared to borderline personality disorder and nonclinical controls. *Molecular Autism*, *14*(1). https://doi.org/10.1186/s13229-023-00580-3

Brenner, J., Pan, Z., Mazefsky, C., Smith, K. A., Gabriels, R., Autism, & Developmental Disorders Inpatient Research, C. (2018). Behavioral Symptoms of Reported Abuse in Children and Adolescents with Autism Spectrum Disorder in Inpatient Settings. *J Autism Dev Disord*, *48*(11), 3727-3735. https://doi.org/10.1007/s10803-017-3183-4

*Bross, L. A., Huffman, J. M., & Hagiwara, M. (2022). Examining the special interest areas of autistic adults with a focus on their employment and mental health outcomes. *Journal of Vocational Rehabilitation*, *57*, 289-305. https://doi.org/10.3233/JVR-221218

*Cappadocia, M. C., Weiss, J. A., & Pepler, D. (2012). Bullying experiences among children and youth with autism spectrum disorders. *J Autism Dev Disord*, *42*(2), 266-277. https://doi.org/10.1007/s10803-011-1241-x

*Courreges, A., Kouros, C. D., Ekas, N. V., & Levy, S. (2023). Remote vs. in-person schooling during the COVID-19 pandemic and internalizing symptoms among children on the autism spectrum. *Research in Autism Spectrum Disorders*, *109*. https://doi:10.1016/j.rasd.2023.102284

*Dababnah, S., Kim, I., & Shaia, W. E. (2021). I am so fearful for him: a mixed-methods exploration of stress among caregivers of Black children with autism. *International Journal of Developmental Disabilities*. https://doi:10.1080/20473869.2020.1870418

*Darazsdi, Z., & Bialka, C. S. (2023). "Oh, you couldn't be autistic": Examining anti-autistic bias and self-esteem in the therapeutic alliance. *Autism: The International Journal of Research & Practice*, *27*(7), 2124-2134. https://doi:10.1177/13623613231154622

*Douglas, S., & Sedgewick, F. (2023). Experiences of interpersonal victimization and abuse among autistic people. *Autism*. https://doi:10.1177/13623613231205630

*Evans, J. A., Krumrei-Mancuso, E. J., & Rouse, S. V. (2023). What You Are Hiding Could Be Hurting You: Autistic Masking in Relation to Mental Health, Interpersonal Trauma, Authenticity, and Self-Esteem. *Autism in Adulthood*. https://doi:10.1089/aut.2022.0115

*Ferrigno, S., Cicinelli, G., & Keller, R. (2022). Bullying in Autism Spectrum Disorder: prevalence and consequences in adulthood. *Journal of Psychopathology*, *28*(3), 127-134. https://doi:10.36148/2284-0249-466

*Fink, E., Olthof, T., Goossens, F., van der Meijden, S., & Begeer, S. (2018). Bullying-related behaviour in adolescents with autism: Links with autism severity and emotional and behavioural problems. *Autism*, *22*(6), 684-692. https://doi.org/10.1177/1362361316686760

*Fisher, M. H., & Taylor, J. L. (2016). Let's talk about it: Peer victimization experiences as reported by adolescents with autism spectrum disorder. *Autism*, *20*(4), 402-411. https://doi.org/https://doi.org/10.1177/1362361315585948

*Gibbs, V., & Pellicano, E. (2023). ‘Maybe we just seem like easy targets’: A qualitative analysis of autistic adults’ experiences of interpersonal violence. *Autism*, *27*(7), 1-14. https://doi.org/10.1177/13623613221150375

*Greenlee, J. L., Winter, M. A., & Marcovici, I. A. (2020). Brief Report: Gender Differences in Experiences of Peer Victimization Among Adolescents with Autism Spectrum Disorder. *J Autism Dev Disord*, *50*(10), 3790-3799. https://doi.org/10.1007/s10803-020-04437-z

*Haruvi-Lamdan, N., Horesh, D., Zohar, S., Kraus, M., & Golan, O. (2020). Autism Spectrum Disorder and Post-Traumatic Stress Disorder: An unexplored co-occurrence of conditions. *Autism*, *24*(4), 884-898. https://doi.org/10.1177/1362361320912143

*Heselton, G. A., Rempel, G. R., & Nicholas, D. B. (2022). “Realizing the problem wasn’t necessarily me”: the meaning of childhood adversity and resilience in the lives of autistic adults. *International Journal of Qualitative Studies on Health and Well-Being*, *17*, 1-10. https://doi.org/10.1080/17482631.2022.2051237

*Holden, R., Mueller, J., McGowan, J., Sanyal, J., Kikoler, M., Simonoff, E., … & Downs, J. (2020). Investigating Bullying as a Predictor of Suicidality in a Clinical Sample of Adolescents with Autism Spectrum Disorder. *Autism Res*, *13*(6), 988-997. https://doi.org/10.1002/aur.2292

*Hong, J., Bishop-Fitzpatrick, L., Smith, L. E., Greenberg, J. S., & Mailick, M. R. (2016). Factors Associated with Subjective Quality of Life of Adults with Autism Spectrum Disorder: Self-Report Versus Maternal Reports. *J Autism Dev Disord*, *46*(4), 1368-1378. https://doi.org/10.1007/s10803-015-2678-0

*Hu, H. F., Liu, T. L., Hsiao, R. C., Ni, H. C., Liang, S. H., Lin, C. F., … & Yen, C. F. (2019). Cyberbullying Victimization and Perpetration in Adolescents with High-Functioning Autism Spectrum Disorder: Correlations with Depression, Anxiety, and Suicidality. *J Autism Dev Disord*, *49*(10), 4170-4180. https://doi.org/10.1007/s10803-019-04060-7

*Kerns, C. M., Lankenau, S., Shattuck, P. T., Robins, D. L., Newschaffer, C. J., & Berkowitz, S. J. (2022). Exploring potential sources of childhood trauma: A qualitative study with autistic adults and caregivers. *Autism*, *26*(8), 1987-1998. https://doi.org/10.1177/13623613211070637

*Kildahl, A. N., & Helverschou, S. B. (2023). Post-traumatic stress disorder and experiences involving violence or sexual abuse in a clinical sample of autistic adults with intellectual disabilities: Prevalence and clinical correlates. *Autism*, *28*(5), 1075-1089. https://doi.org/10.1177/13623613231190948

*Leung, P. W. S., Li, S. X., Holroyd, E. A., Tsang, C. S. O., & Wong, W. C. W. (2023). Online social media poses opportunities and risks in autistic youth: implications for services from a qualitative study. *Frontiers in Psychiatry*, *14*. https://doi.org/10.3389/fpsyt.2023.959846

*Lin, P. C., Peng, L. Y., Hsiao, R. C., Chou, W. J., & Yen, C. F. (2020). Teacher Harassment Victimization in Adolescents with High-Functioning Autism Spectrum Disorder: Related Factors and Its Relationships with Emotional Problems. *Int J Environ Res Public Health*, *17*(11). https://doi.org/10.3390/ijerph17114057

*Liu, T. L., Wang, P.-W., Hsiao, R. C., Ni, H.-C., Liang, S. H., Lin, C.-F., … & Yen, C.-F. (2022). Multiple types of harassment victimization in adolescents with autism spectrum disorder: Related factors and effects on mental health problems. *Journal of the Formosan Medical Association*, *121*, 2161-2171. https://doi.org/10.1016/j.jfma.2022.03.005

*Lu, H.-H., Chen, D.-R., & Chou, A.-K. (2022). The school environment and bullying victimization among seventh graders with autism spectrum disorder: a cohort study. *Child and Adolescent Psychiatry and Mental Health*, *16*(1), 1-9. https://doi.org/10.1186/s13034-022-00456-z

*Marion, A., Bowman, K., Thomas, G., & Harrison, A. J. (2023). A mixed method examination: how stigma experienced by autistic adults relates to metrics of social identity and social functioning. *Frontiers in Psychiatry*, *14*. https://doi.org/10.3389/fpsyt.2023.1243618

*Mayes, S. D., Gorman, A. A., Hillwig-Garcia, J., & Syed, E. (2013). Suicide ideation and attempts in children with autism. *Research in Autism Spectrum Disorders*, *7*, 109-119. https://doi.org/10.1016/j.rasd.2012.07.009

*McClemont, A. J., Morton, H. E., Gillis, J. M., & Romanczyk, R. G. (2020). Brief report: Predictors of school refusal due to bullying in children with autism spectrum disorder and attention-deficit/hyperactivity disorder. *Journal of Autism and Developmental Disorders*. https://doi.org/10.1007/s10803-020-04640-y

*McDonnell, C. G., Andrzejewski, T., & Dike, J. (2021). Intergenerational trauma: Parental PTSD and parent-reported child abuse subtypes differentially relate to admission characteristics in the autism inpatient collection. *Autism Research : Official Journal of the International Society for Autism Research*, *15*(4), 665-676. https://doi.org/10.1002/aur.2669

*Ochi, M., Kawabe, K., Ochi, S., Miyama, T., Horiuchi, F., & Ueno, S. I. (2020). School refusal and bullying in children with autism spectrum disorder. *Child Adolesc Psychiatry Ment Health*, *14*, 17. https://doi.org/10.1186/s13034-020-00325-7

*Pearson, A., Rees, J., & Forster, S. (2022). ‘‘This was just how this friendship worked’’: Experiences of interpersonal victimization among autistic adults. *Autism in Adulthood*, *4*(2), 1-10. https://doi.org/10.1089/aut.2021.0035

*Pearson, A., Rose, K., & Rees, J. (2023). ‘I felt like I deserved it because I was autistic’: Understanding the impact of interpersonal victimisation in the lives of autistic people. *Autism*, *27*(2), 500-511. https://doi.org/10.1177/13623613221104546

*Pfeffer, R. D. (2016). Childhood victimization in a national sample of youth with autism spectrum disorders. *Journal of Policy and Practice in Intellectual Disabilities*, *13*(4), 311-319. https://doi.org/10.1111/jppi.12203

*Reuben, K. E., Stanzione, C. M., & Singleton, J. L. (2021). Interpersonal trauma and posttraumatic stress in autistic adults. *Autism in Adulthood*, *3*(3), 247-256. https://doi.org/10.1089/aut.2020.0073

*Rodriguez, G., Drastal, K., & Hartley, S. L. (2021). Cross-lagged model of bullying victimization and mental health problems in children with autism in middle to older childhood. *Autism*, *25*(1), 90-101. https://doi.org/10.1177/1362361320947513

*Rothman, E. F., & Graham Holmes, L. (2022). Using formative research to develop HEARTS: A curriculumbased healthy relationships promoting intervention for individuals on the autism spectrum. *Autism*, *26*(1), 160-168. https://doi.org/10.1177/13623613211024521

*Sedgewick, F., Hill, V., & Pellicano, E. (2019). ‘It’s different for girls’: Gender differences in the friendships and conflict of autistic and neurotypical adolescents. *Autism*, *23*(5), 1119-1132. https://doi.org/10.1177/1362361318794930

Tan, D. W., Rabuka, M., Haar, T., & Pellicano, E. (2023). ‘It’s a symbolic violence’: Autistic people’s experiences of discrimination at universities in Australia. *Autism*. https://doi.org/10.1177/13623613231219744

*Toseeb, U., McChesney, G., Oldfield, J., & Wolke, D. (2020). Sibling bullying in middle childhood is associated with psychosocial difficulties in early adolescence: The case of individuals with autism spectrum disorder. *J Autism Dev Disord*, *50*(5), 1457-1469. https://doi.org/10.1007/s10803-019-04116-8

*Toseeb, U., McChesney, G., & Wolke, D. (2018). The prevalence and psychopathological correlates of sibling bullying in children with and without autism spectrum disorder. *J Autism Dev Disord*, *48*(7), 2308-2318. https://doi.org/10.1007/s10803-018-3484-2

*Ung, D., McBride, N., Collier, A., Selles, R., Small, B., Phares, V., & Storch, E. (2016). The relationship between peer victimization and the psychological characteristics of youth with autism spectrum disorder. *Research in Autism Spectrum Disorders*, *32*, 70-79. https://doi.org/10.1016/j.rasd.2016.09.002

*Weiss, J. A., Cappadocia, M. C., Tint, A., & Pepler, D. (2015). Bullying victimization, parenting stress, and anxiety among adolescents and young adults with autism spectrum disorder. *Autism Res*, *8*(6), 727-737. https://doi.org/10.1002/aur.1488

*Wright, M. F. (2018). Cyber Victimization and Depression among Adolescents with Autism Spectrum Disorder: The Buffering Effects of Parental Mediation and Social Support. *J Child Adolesc Trauma*, *11*(1), 17-25. https://doi.org/10.1007/s40653-017-0169-5

*Yoshikawa, H., Kitamura, S., Matsuoka, K., Takahashi, M., Ishida, R., Kishimoto, N., … & Makinodan, M. (2022). Adverse childhood experience is associated with disrupted white matter integrity in autism spectrum disorder: A diffusion tensor imaging study. *Original Research*, *12*, 1-9. https://doi.org/10.3389/fpsyt.2021.823260

Zablotsky, B., Bradshaw, C. P. A., C. , & Law, P. A. (2013). The association between bullying and the psychological functioning of children with autism spectrum disorders. *Journal of Developmental and Behavioral Pediatrics*, *34*(1), 1-8. https://doi.org/10.1097/DBP.0b013e31827a7c3a

Zablotsky, B. B., Anderson, C., & Law, P. A. (2013). The association between bullying and the psychological functioning of children with autism spectrum disorders. *Journal of Developmental and Behavioral Pediatrics*, *34*(1), 1-8. https://doi.org/10.1097/DBP.0b013e31827a7c3a

Zeedyk, S. M., Rodriguez, G., Tipton, L. A., Baker, B. L., & Blacher, J. (2014). Bullying of youth with autism spectrum disorder, intellectual disability, or typical development: Victim and parent perspectives. *Res Autism Spectr Disord*, *8*(9), 1173-1183. https://doi.org/10.1016/j.rasd.2014.06.001
